# Computational and stability analysis of Ebola virus epidemic model with piecewise hybrid fractional operator

**DOI:** 10.1371/journal.pone.0298620

**Published:** 2024-04-16

**Authors:** Kottakkaran Sooppy Nisar, Muhammad Farman, Khadija Jamil, Ali Akgul, Saba Jamil

**Affiliations:** 1 Department of Mathematics, College of Arts and Sciences, Prince Sattam bin Abdulaziz University, Wadi Aldawaser, Saudi Arabia; 2 Faculty of Arts and Sciences, Department of Mathematics, Near East University, Nicosia, Northern Cyprus, Turkey; 3 Department of Computer Science and Mathematics, Lebanese American University, Beirut, Lebanon; 4 Institute of Mathematics, Khwaja Fareed University of Engineering and Information Technology, Rahim Yar Khan, Pakistan; 5 Faculty of Arts and Science, Department of Mathematics, Siirt University, Siirt, Turkey; Adana Alparslan Türkeş Science and Technology University: Adana Alparslan Turkes Bilim ve Teknoloji Universitesi, TURKEY

## Abstract

In this manuscript, we developed a nonlinear fractional order Ebola virus with a novel piecewise hybrid technique to observe the dynamical transmission having eight compartments. The existence and uniqueness of a solution of piecewise derivative is treated for a system with Arzel’a-Ascoli and Schauder conditions. We investigate the effects of classical and modified fractional calculus operators, specifically the classical Caputo piecewise operator, on the behavior of the model. A model shows that a completely continuous operator is uniformly continuous, and bounded according to the equilibrium points. The reproductive number *R*_0_ is derived for the biological feasibility of the model with sensitivity analysis with different parameters impact on the model. Sensitivity analysis is an essential tool for comprehending how various model parameters affect the spread of illness. Through a methodical manipulation of important parameters and an assessment of their impact on *R*_*o*_, we are able to learn more about the resiliency and susceptibility of the model. Local stability is established with next Matignon method and global stability is conducted with the Lyapunov function for a feasible solution of the proposed model. In the end, a numerical solution is derived with Newton’s polynomial technique for a piecewise Caputo operator through simulations of the compartments at various fractional orders by using real data. Our findings highlight the importance of fractional operators in enhancing the accuracy of the model in capturing the intricate dynamics of the disease. This research contributes to a deeper understanding of Ebola virus dynamics and provides valuable insights for improving disease modeling and public health strategies.

## 1 Introduction

Ebola virus disease (EVD), initially detected in Africa, is an infrequently occurring and potentially fatal condition. Nonhuman primates and humans are both affected by EVD. In the Democratic Republic of the Congo, near to the Ebola River, the Ebola virus was first discovered in 1976. Since then, the virus has periodically caused epidemics in different African countries [1, 2]. Scientists are unsure about the exact virus’s origin. Rachah et al. [3] analyzed a straightforward mathematical model that portrayed the 2014 Ebola outbreak in Liberia. Later, the mathematical model was validated through the use of computer simulations and previous data from the World Health Organization (WHO). Additionally, a novel mathematical model was developed which incorporates immunization rates. To compare the classical and fractional SEIR epidemic Ebola virus models with actual data the reports of the World Health Organization from March 27, 2014, [4] were utilized. Two mathematical models were assessed in order to elucidate the ongoing spread of the Ebola virus in West Africa [5]. Researchers have utilized mathematical modeling to forecast the transmission of viruses, including the Ebola virus, among humans. Several classical mathematical approaches, like the SI model, SIR model, SEIR model, SEIRD model, and SEIRHD model, have been employed to describe the disease caused by the Ebola virus (EVD) [6, 7]. To combat EVD, the World Medical Association has developed specialized medicines for treating the Ebola virus. Modeling EVD epidemics involves employing quantitative methods and analyzing the reproduction rate of Ebola outbreaks. Furthermore, researchers have examined using the demographic information on Ebola risk factors and viral transmission house- hold structured epidemic model, leading to significant predictions, valuable insights, and the disclosure of pertinent personal and genomic data related to EVD through the application of mathematical models. In their research, Ismail et al. [8] explored the Ebola virus, which falls under the category of negative sense RNA viruses with a single strand. This virus causes the deadly, hemorrhagic, and extremely contagious Ebola virus disease (EVD). EVD stands as one of the most alarming health threats, resulting in numerous fatalities.

The Sinc Legendre collocation technique was employed to address a nonlinear fractional model of the Caputo sense Ebola Virus Disease (EVD) [9]. By utilizing the Atangana Baleanu and Caputo (ABC) derivative the EVD fractional model was investigated and solutions were obtained through the application of fixed point theory [10]. Their research highlighted the importance of the dynamics of epidemic infection rate and reproduction capacity. The significance and broad applicability of fractional models [11] and nonlinear dynamical systems through the differential transform [12]. In recent studies researchers have explored various aspects of fractional analysis in different contexts. The examination of the migration effect in plant-pathogen-herbivore interactions was specifically conducted through the utilization of piecewise fractional analysis [13]. Another study examined the fractional model of COVID-19 by means of piecewise global operators [14]. Furthermore, the dynamics of leptospirosis disease have been explored within the confines of piecewise classical-global and classical-fractional operators [15]. Studies have explored generalized fractal-fractional order problems focusing on non-singular Mittag-Leffler kernels in one instance [16]. Another investigation has been conducted on a mathematical model for financial bubbles employing a fractal-fractional Caputo derivative [17]. The Herz-Tuckwill model [18] to provide a structure for examining T-cytotoxic lymphocyte responses to the Ebola virus. To test the equilibrium’s overall stability, they next used the LaSalle invariance rule and a powerful Lyapunov function. A supervised learning-based computer paradigm [19] to examine a nonlinear SEIR model of EVD. In the work of [20], a mathematical model known as the fractional SIRI model which incorporates time delay is investigated. Additionally, [21] examines an SIR model that utilizes the Mittag-Leffler fractional derivative. Prior studies have explored the dynamic characteristics of a discrete-time SIR epidemic model [22]. In their study of the nonlinear SEIR-HVQD model of EVD, Meta- heuristic Ebola [23] optimization search technique. To analyse how the Ebola virus disease (EVD) spreads in a particular area, [24] created an epidemic model. The model considered a number of preventative measures, including vaccination, public awareness campaigns, early diagnostic programs, improved hospital sanitation, isolation of sick individuals, and geographic movement restrictions. The researchers wanted to learn more about how these measures collectively affect EVD transmission and control in the region, so they incorporated them into their model to that end. They employed optimal control theory to identify an effective prevention strategy that reduces the number of sick people, considering operational constraints. In recent studies, a new adaptive nonlinear numerical approach for handling singular and stiff differential problems was presented [25]. Additionally, mathematical dynamics for HIV infections were investigated taking into account public awareness and the detectability of viral load [26]. Furthermore, a study of COVID-19 dynamics in Thailand has been carried out [27]. Furthermore, a thorough investigation has been carried out on the application of the Caputo fractional-order derivative in the dynamical analysis of a generalized tumor model [28]. Finally, a study has been conducted [29] on dual-wave solutions for the Kadomtsev-Petviashvili model that include second-order temporal and spatial-temporal dispersion factors.

Various operators such as fractal derivatives, non-integer order derivatives utilizing kernels with singularity and non-singularity, fractal-fractional operators and other derivative operators have been introduced to explore crossover phenomena [30, 31]. Despite incorporating randomness through stochastic equations, the issue of crossover dynamics persists. Many models, encompassing KdV-CDG equation, Schrodinger systems, and Black-Scholes problems, exhibit these characteristics [32–34]. Classical and global piecewise differentiation and integration have emerged as novel approaches [35] to tackle such challenges. The discussion delves into addressing these challenges by applying piecewise derivatives and integrations supplemented with various illustrative examples. This becomes essential especially given the struggles faced by exponential and Mittag-Leffler mappings in fractional calculus when attempting to ascertain crossover timings. Through the elimination of discontinuities and ensuring the system’s differentiability we can characterize the system’s continuity within each interval by employing the piecewise derivative of a function. Furthermore, there is an option to extend the derivative and function as a global operator by incorporating a singular kernel in the second interval grounded in the power law. This method thoroughly explains the system’s dynamics over the interval (0, 1]. We will use piecewise differential operators to simulate the EVD in this work. Integrating classical and modified fractional calculus operators into the Ebola model allows for a more nuanced and accurate depiction of the disease dynamics, thereby facilitating a better understanding of the virus’s behavior and its implications for public health strategies.

There are eight sections in this paper. Section 1 is an introduction, while section 2 elucidates various fractional-order derivatives that can be employed to solve the epidemiological model. The suggested models positively invariant, equilibrium points, reproductive potential and sensitivity analysis in section 3. The stability evaluation of the proposed model is done in section 4 and includes Lyapunov stability. The existence and uniqueness of a system of solutions to the Caputo piecewise model is verified using the fixed point theory in section 5. In section 6, we look at Caputo numerical system piecewise. Sections 7 and 8 discuss the results and conclusion, respectively.

## 2 Basic concepts of piecewise fractional operator

Furthermore, as outlined in the paper [36], the fractional-time derivative demonstrates a noteworthy relationship with memory and finds applications in diverse fields such as physics [36], biology and ecology [37, 38], economy [39] and chemistry [40]. This emphasizes the derivative’s enormous relevance in epidemiology and other fields. Indeed, the suggestion is made that the memory functional serves as the kernel for the fractional-order derivative and the order of the time derivative highlights the rate of memory retention. Understanding the dynamical characteristics of systems in general and epidemiological models in particular can be done using the fractional calculus. The ability to measure memory using fractional-order derivative definition is shown in [41]. In the case of infectious diseases, we can emphasize that this memory can be seen in how individuals take precautions, use treatments, vaccinations, and protective gear to keep themselves from contracting the disease. The order of the derivative emphasizes how people take precautions as a population, whereas for integer derivatives there is no precaution against contracting the disease and as the fractional derivative rate decreases the precautions increase.

**Definition 1** Let ℑ(*t*) be a function that is not always differentiable. Let *η* be a fractional order and 0 < *η* < *T*_1_. According to [42], a fractal fractional derivative (FFD) of order 0 < *η* < *T*_1_ with Caputo is

D0Ctηℑ(t)=1Γ(-η+1)∫0t(t-α)-ηℑ(α)dα. (1)

**Definition 2** Let ℑ(*t*) be a function that is not always differentiable. Let *η* be a fractional order and 0 < *η* < *T*_1_. According to [42], a fractal fractional integral (FFI) of order 0 < *η* < *T*_1_ with Caputo is defined as

J0Ctηℑ(t)=1Γ(η)∫0t(t-α)η-1dα. (2)

**Definition 3** Suppose that it is possible to differentiate ℑ(*t*). The piecewise derivative with a power law kernel and a classical and fractional derivative is defined as [35].

D0PCtηℑ(t)={ℑ′(t),0<t<t1,D0Ctηℑ(t),0<t<t2, (3)

Where D0PCtη stands for the Caputo fractional derivative on *t*_1_ < *t* < *t*_2_ and the classical derivative on 0 < *t* < *t*_1_.

**Definition 4** The traditional Caputo integration is as shown in reference [35] if we suppose that ℑ(*t*) is a differentiable function.

D0PCtηℑ(t)={∫0tℑ(α)dα,0<t<t1,1Γ(η)∫0t(t-α)η-1dα,t1<t<t2. (4)

Where D0PCtη stands for the Caputo fractional derivative on *t*_1_ < *t* < *t*_2_ and the classical derivative on 0 < *t* < *t*_1_.

**Lemma 1** In [35], an equation featuring a piecewise derivative can exhibit the following solutions.

D0PCtηℑ(t)=K(t,ℑ(t)), (5)

is

$$\begin{array}{c} ℑ\left(t\right)=\{\begin{array}{c} {ℑ}_{0}+\int_{0}^{t}ℑ\left(\alpha \right)d\alpha ,0<t<t_{1},\\ ℑ\left(t_{1}\right)+\frac{1}{\Gamma (\eta )}\int_{0}^{t}{\left(t-\alpha \right)}^{\eta -1}d\alpha ,t_{1}<t<t_{2}. \end{array} \end{array}$$ (6)

## 3 Fractional order model of ebola virus model

The model of the Ebola virus was presented in [43]. The total N population is divided into eight mutually exclusive classes: susceptible population (S), exposed population (E), infected population (I), hospitalized population (H), asymptomatic but still infectious population (R), a population which is dead but not interred (D), interred population (B), and fully recovered population (C). The model utilizes piecewise Caputo derivatives to describe the rate of change of these compartments over time, incorporating memory effects and non-local behaviors that differ from traditional integer-order models. This approach allows for more realistic representations of disease spread dynamics, accounting for delays, memory-dependent effects, and discontinuities in the disease progression, offering a more nuanced view of the epidemic’s behavior compared to conventional models that use integer-order derivatives.

{D0PCtηS(t)=μN-βiNSI-βhNSH-βdNSD-βγNSR-μS,D0PCtηE(t)=βiNSI+βhNSH+βdNSD+βγNSR-σE-μE,D0PCtηI(t)=σE-(γ1+ε+τ+μ)I,D0PCtηR(t)=γ1I-γ2H-(γ3+μ)R,D0PCtηD(t)=εI-(δ1+ξ)D,D0PCtηH(t)=τI-(γ2+δ2+μ)H,D0PCtηB(t)=δ1D+δ2H-ξB,D0PCtηC(t)=γ3R-μC, (7)

with the initial conditions

$$\begin{array}{c} S(0)\geq 0\;,\;\;\;E(0)\geq 0\;,\;\;\;I(0)\geq 0\;,\;\;\;R(0)\geq 0\;,\;\;\;D(0)\geq 0\;,\;\;\;H(0)\geq 0\;,\;\;\;B(0)\geq 0\;,\;\;\;C(0)\geq 0\;. \end{array}$$

The overall population is consequently established by

$$\begin{array}{c} N(t)=S(t)+E(t)+I(t)+R(t)+D(t)+H(t)+B(t)+C(t). \end{array}$$ (8)

**Fig 1:**
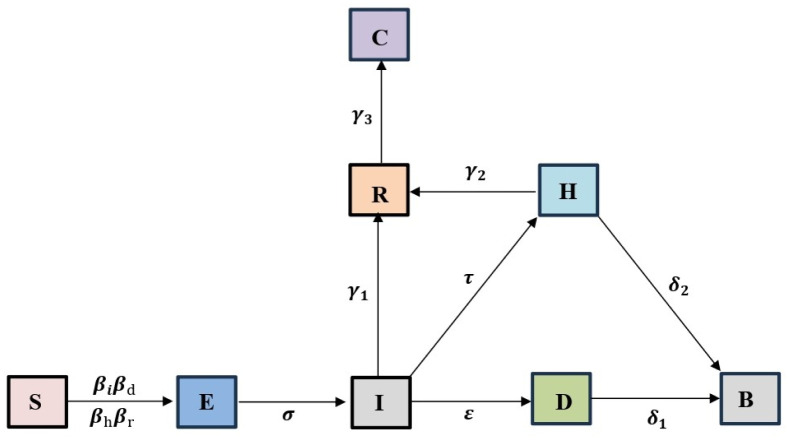
Schematic diagram of Ebola model.

The parameters of the model descriptions and values are given in Table 1. The model schematic diagram is depicted in Fig 1.

**Table 1:** The Ebola virus model parameters are displayed.

Symbol	Description [43]	Value
*σ*	After-Exposure Infectiousness Rate	$\frac{1}{11.4}$
*μ*	Death rate	$\frac{14}{1000}$
*β*_*i*_	Relation between infectious and susceptible.	0.14
*β*_*d*_	Link between infective and dead individuals	0.40
*β*_*h*_	Relation between infective and hospitalized individuals	0.29
*β*_*r*_	Relation between infective and asymptomatic individuals	0.185
*γ*_1_	Rate at which an infection becomes asymptomatic	$1=\frac{1}{10}$
*ϵ*	Fatality rate	$1=\frac{9}{6}$
*δ*_1_	Rate at which an dead becomes interred	1 = 2
*δ*_2_	Rate at which hospitalized becomes interred	$1=\frac{4}{6}$
*γ*_2_	Rate at which hospitalized becomes asymptomatic	1 = 5
*τ*	Rate at which infectious class becomes hospitalized class	1 = 5
*γ*_3_	Rate at which asymptomatic class becomes completely recovered class	$1=\frac{1}{30}$
*ξ*	Incineration rate	$14=\frac{1000}{1}$

### 3.1 Positively invariant region

In this section, we demonstrate that the closed set

$$\gamma_{l}=\{(S,E,I,R,D,H,B,C)\in R_{+}^{8}:0\leq N\},$$

provides the system (7) positively invariant feasibility region.

**Lemma 3.1** The solutions of system (7) are non-negative and bounded, if they start in *γ*_*l*_.

**Proof:** From the model (7), we find that

{D0PCtηS(t)|S=0=μN≥0,D0PCtηE(t)|E=0=βiNSI+βhNSH+βdNSD+βγNSR≥0,D0PCtηI(t)|I=0=σE≥0,D0PCtηR(t)|R=0=γ1I+γ2H≥0,D0PCtηD(t)|D=0=εI≥0,D0PCtηH(t)|H=0=τI≥0,D0PCtηB(t)|B=0=δ1D+δ2H≥0,D0PCtηC(t)|C=0=γ3R≥0. (9)

Thus the sub-population *S*(*t*), *E*(*t*), *I*(*t*), *R*(*t*), *D*(*t*), *B*(*t*) and *C*(*t*) are non-negative. As *S*(*t*) + *E*(*t*) + *I*(*t*) + *R*(*t*)+ *D*(*t*) + *B*(*t*) + *C*(*t*) = *N*, where the total population *N* is considered to be constant, each subpopulation lies in [0, *N*]. Hence the sub populations *S*(*t*), *E*(*t*), *I*(*t*), *R*(*t*), *D*(*t*), *B*(*t*) and *C*(*t*) are bounded as well.

### 3.2 Equilibrium points and *R*_0_

Disease-free points of the above model are *E*_0_ = (*S*^−^, *E*^−^, *I*^−^, *R*^−^, *D*^−^, *H*^−^, *B*^−^, *C*^−^)=(*N*, 0, 0, 0, 0, 0, 0, 0) however, the endemic equilibrium point is

$$\begin{array}{c} E^{*}=(S^{+},E^{+},I^{+},R^{+},D^{+},H^{+},B^{+},C^{+})\\ =(\begin{array}{c} \frac{N\mu q_{1}q_{2}q_{3}q_{4}+Nq_{1}q_{2}q_{3}q_{4}\sigma }{\beta_{r}\gamma_{1}q_{3}q_{4}\sigma -\beta_{r}\gamma_{2}q_{3}\sigma \tau +\beta_{i}q_{2}q_{3}q_{4}\sigma +\beta_{d}q_{2}q_{4}\sigma \varepsilon +N\beta_{h}q_{2}q_{3}\sigma \tau }\\ ,\frac{\mu \Theta }{\sigma \Omega },\frac{\mu \Theta }{q_{1}\Omega },\frac{\mu \Theta_{1}}{\Xi },\frac{\mu \varepsilon \Theta }{q_{1}q_{3}\Omega },\frac{\mu \tau \Theta }{\Omega_{1}},\frac{\mu \Theta (q_{3}\sigma_{2}\tau +q_{4}\sigma_{1}\varepsilon )}{q_{3}\xi \Omega_{1}},\frac{\gamma_{3}\Theta_{1}}{\Xi } \end{array}) \end{array}$$

where

$$\begin{array}{c} \Theta =N^{2}\beta_{h}q_{2}q_{3}\sigma \tau +N\beta_{r}\gamma_{1}q_{3}q_{4}\sigma -N\beta_{r}\gamma_{2}q_{3}\sigma \tau +N\beta_{i}q_{2}q_{3}q_{4}\sigma +N\beta_{d}q_{2}q_{4}\sigma \varepsilon -N\mu q_{1}q_{2}q_{3}q_{4}-Nq_{1}q_{2}q_{3}q_{4}\sigma , \end{array}$$

$$\begin{array}{cc} \Theta_{1} & =N\beta_{r}\gamma_{1}^{2}q_{3}q_{4}^{2}\sigma +N\beta_{r}\gamma_{2}^{2}q_{3}\sigma \tau^{2}+N\beta_{i}\gamma_{1}q_{2}q_{3}q_{4}^{2}\sigma +N\beta_{d}\gamma_{1}q_{2}q_{4}^{2}\sigma \varepsilon -N\gamma_{1}\mu q_{1}q_{2}q_{3}q_{4}^{2}\\ & -N\gamma_{1}q_{1}q_{2}q_{3}q_{4}^{2}\sigma -N^{2}\beta_{h}\gamma_{2}q_{2}q_{3}\sigma \tau^{2}-2N\beta_{r}\gamma_{1}\gamma_{2}q_{3}q_{4}\sigma \tau -N\beta_{i}\gamma_{2}q_{2}q_{3}q_{4}\sigma \tau \\ & -N\beta_{d}\gamma_{2}q_{2}q_{4}\sigma \tau \varepsilon +N\gamma_{2}\mu q_{1}q_{2}q_{3}q_{4}\tau +N\gamma_{2}q_{1}q_{2}q_{3}q_{4}\sigma \tau +N^{2}\beta_{h}\gamma_{1}q_{2}q_{3}q_{4}\sigma \tau , \end{array}$$

$$\begin{array}{c} \Omega =\beta_{r}\gamma_{1}\mu q_{3}q_{4}-\beta_{r}\gamma_{2}\mu q_{3}\tau +\beta_{r}\gamma_{1}q_{3}q_{4}\sigma -\beta_{r}\gamma_{2}q_{3}\sigma \tau +\beta_{i}\mu q_{2}q_{3}q_{4}\\ +\beta_{d}\mu q_{2}q_{4}\varepsilon +\beta_{i}q_{2}q_{3}q_{4}\sigma +\beta_{d}q_{2}q_{4}\sigma \varepsilon +N\beta_{h}\mu q_{2}q_{3}\tau +N\beta_{h}q_{2}q_{3}\sigma \tau , \end{array}$$

$$\begin{array}{c} \Omega_{1}=\beta_{r}\gamma_{1}\mu q_{1}q_{3}q_{4}^{2}+\beta_{r}\gamma_{1}q_{1}q_{3}q_{4}^{2}\sigma +\beta_{i}\mu q_{1}q_{2}q_{3}q_{4}^{2}+\beta_{d}\mu q_{1}q_{2}q_{4}^{2}\varepsilon +\beta_{i}q_{1}q_{2}q_{3}q_{4}^{2}\sigma +\beta_{d}q_{1}q_{2}q_{4}^{2}\sigma \varepsilon \\ -\beta_{r}\gamma_{2}\mu q_{1}q_{3}q_{4}\tau -\beta_{r}\gamma_{2}q_{1}q_{3}q_{4}\sigma \tau +N\beta_{h}\mu q_{1}q_{2}q_{3}q_{4}\tau +N\beta_{h}q_{1}q_{2}q_{3}q_{4}\sigma \tau , \end{array}$$

$$\begin{array}{c} \Xi =\beta_{i}\mu q_{1}q_{2}^{2}q_{3}q_{4}^{2}+\beta_{d}\mu q_{1}q_{2}^{2}q_{4}^{2}\varepsilon +\beta_{i}q_{1}q_{2}^{2}q_{3}q_{4}^{2}\sigma +\beta_{d}q_{1}q_{2}^{2}q_{4}^{2}\sigma \varepsilon +\beta_{r}\gamma_{1}\mu q_{1}q_{2}q_{3}q_{4}^{2}\\ +\beta_{r}\gamma_{1}q_{1}q_{2}q_{3}q_{4}^{2}\sigma +N\beta_{h}\mu q_{1}q_{2}^{2}q_{3}q_{4}\tau +N\beta_{h}q_{1}q_{2}^{2}q_{3}q_{4}\sigma \tau \\ -\beta_{r}\gamma_{2}\mu q_{1}q_{2}q_{3}q_{4}\tau -\beta_{r}\gamma_{2}q_{1}q_{2}q_{3}q_{4}\sigma \tau . \end{array}$$

The basic reproduction number (which represents the average number of secondary infections generated by a single infection in a susceptible population) can be computed for model (7) using the next-generation matrix method as described in reference [44], we calculate *R*_*o*_ in the following manner: by taking into account the newly occurring infections and the transfer matrices, we evaluate the corresponding Jacobian matrices F and V at the Disease-Free Equilibrium (DFE).

$$F=[\begin{array}{ccccc} 0 & \beta_{i} & \beta_{r} & \beta_{d} & \beta_{h}\\ 0 & 0 & 0 & 0 & 0\\ 0 & 0 & 0 & 0 & 0\\ 0 & 0 & 0 & 0 & 0\\ 0 & 0 & 0 & 0 & 0 \end{array}]$$

$$V=[\begin{array}{ccccc} \sigma +\mu & 0 & 0 & 0 & 0\\ -\sigma & \gamma_{1}+\varepsilon +\tau +\mu & 0 & 0 & 0\\ 0 & -\gamma_{1} & \gamma_{3}+\mu & 0 & \gamma_{2}\\ 0 & -\xi & 0 & \delta_{1}+\xi & 0\\ 0 & -\tau & 0 & 0 & \gamma_{2}+\delta_{2}+\mu \end{array}]$$

The reproductive number is defined as the spectral radius of the next generation matrix, specifically represented as *FV*^−1^.

$$FV^{-1}=[\begin{array}{ccccc} {A_{1}}_{1} & {A_{1}}_{2} & {A_{1}}_{3} & {A_{1}}_{4} & {A_{1}}_{5}\\ 0 & 0 & 0 & 0 & 0\\ 0 & 0 & 0 & 0 & 0\\ 0 & 0 & 0 & 0 & 0\\ 0 & 0 & 0 & 0 & 0 \end{array}]$$

where

$$A_{11}=\frac{q_{3}\beta_{i}\sigma q_{4}q_{5}+q_{3}\beta_{r}\left(q_{5}\gamma_{1}+\tau \gamma_{2}\right)+\beta_{d}\varepsilon \sigma q_{4}q_{5}+q_{3}\beta_{h}\tau \sigma q_{4}}{q_{1}q_{2}q_{3}q_{4}q_{5}},$$

$$A_{12}=\frac{\beta_{r}}{q_{4}}+\frac{\beta_{r}\left(q_{5}\gamma_{1}+\tau \gamma_{2}\right)}{q_{2}q_{3}q_{4}}+\frac{\beta_{d}\varepsilon }{q_{2}q_{3}}+\frac{\beta_{h}\tau }{q_{2}q_{5}},$$

$$A_{13}=\frac{\beta_{r}}{q_{4}},$$

$$A_{14}=\frac{\beta_{d}}{q_{3}},$$

$$A_{15}=\frac{\beta_{r}\gamma_{2}}{q_{4}q_{5}}+\frac{\beta_{h}}{q_{5}},$$

with *q*_1_ = *γ*_1_ + *ε* + *τ* + *μ*, *q*_2_ = *γ*_3_ + *μ*, *q*_3_ = *δ*_1_ + *ξ*, *q*_4_ = *γ*_2_ + *δ*_2_ + *μ* and *q*_5_ = *σ* + *μ*. Consequently, the fundamental reproduction number *R*_0_ is given by

$$\begin{array}{c} R_{0}=\frac{\sigma }{q_{1}q_{2}q_{3}q_{4}q_{5}}[q_{3}\left(\beta_{i}\left(\gamma_{3}q_{4}+\mu \left(\gamma_{2}+\delta_{2}\right)+\mu^{2}\right)+\beta_{r}\left(\gamma_{1}q_{4}+\gamma_{2}\tau \right)+\beta_{h}\tau q_{2}\right)+\beta_{d}\varepsilon (\left(\gamma_{3}q_{4}+\mu \left(\gamma_{2}+\delta_{2}\right)\right)+\mu^{2}]. \end{array}$$ (10)

**Fig 2:**
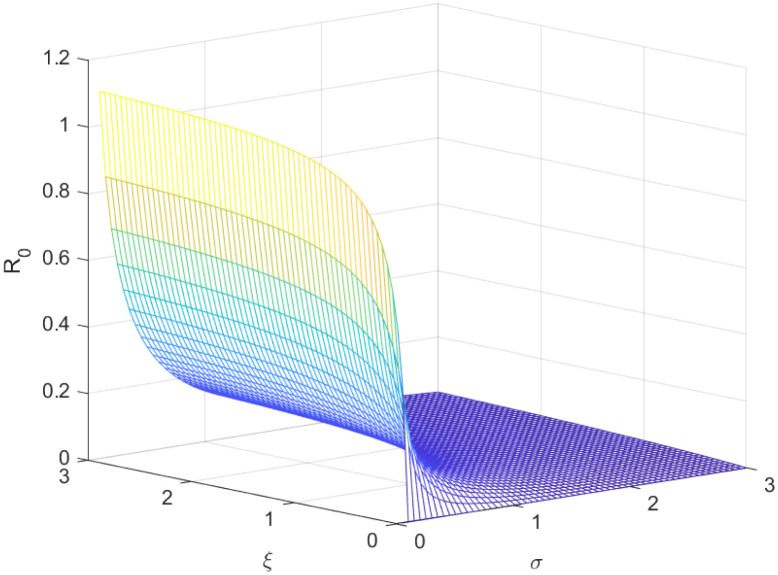
Effect of *ξ* and *σ* on reproductive number.

**Fig 3:**
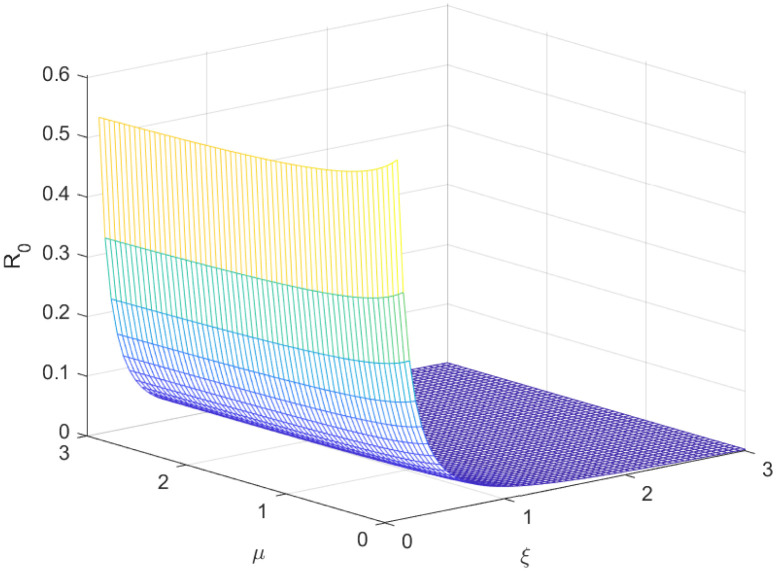
Effect of *μ* and *ξ* on reproductive number.

**Fig 4:**
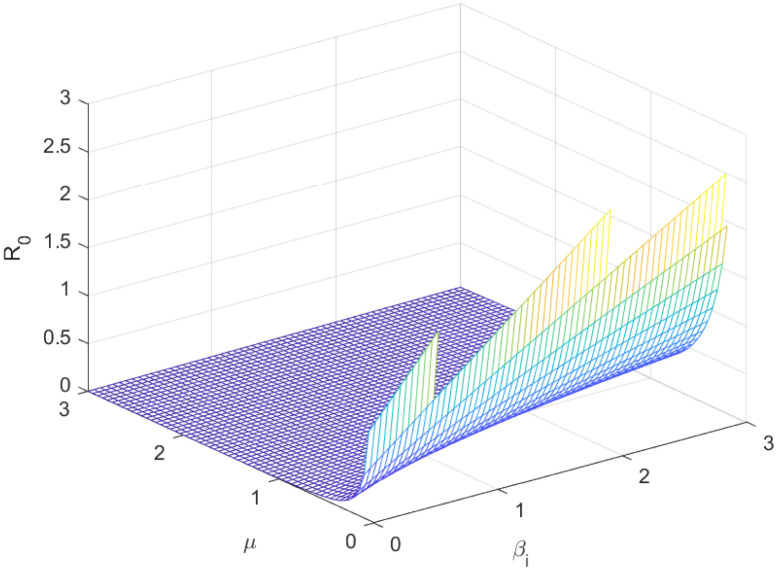
Effect of *μ* and *β*_*i*_ on reproductive number.

**Fig 5:**
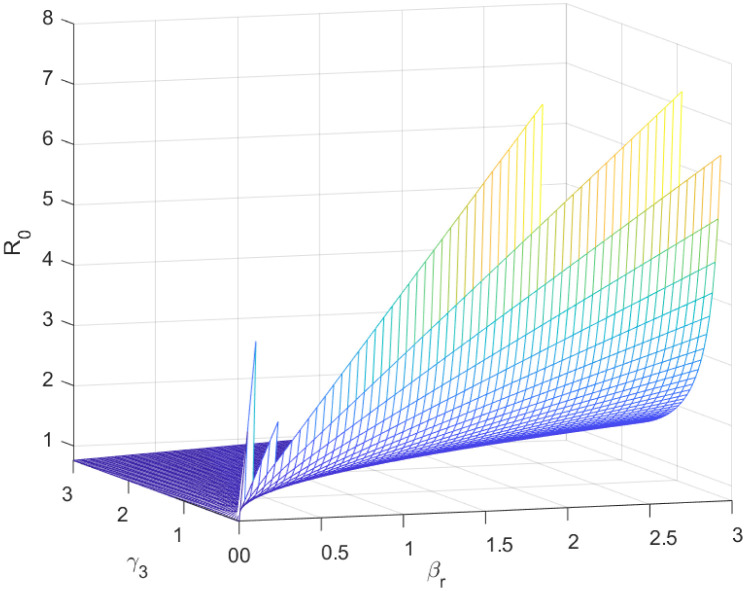
Effect of *γ*_3_ and *β*_*r*_ on reproductive number.

**Fig 6:**
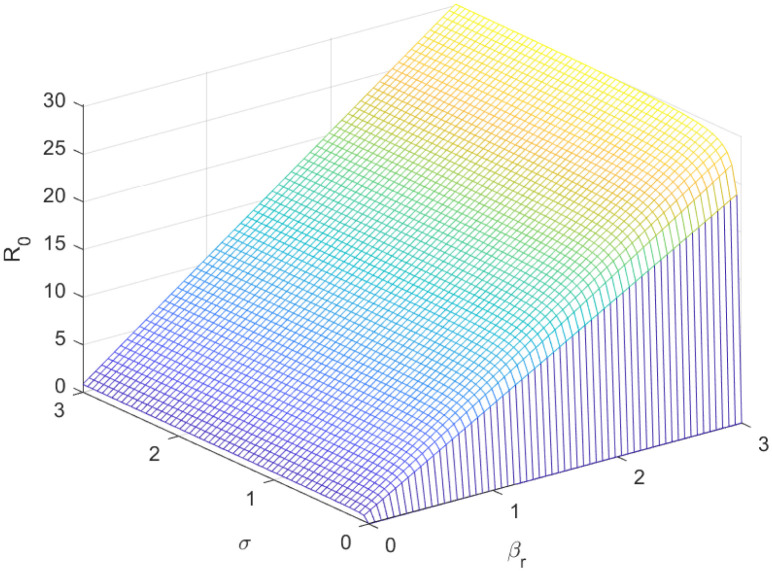
Effect of *σ* and *β*_*r*_ on reproductive number.

**Fig 7:**
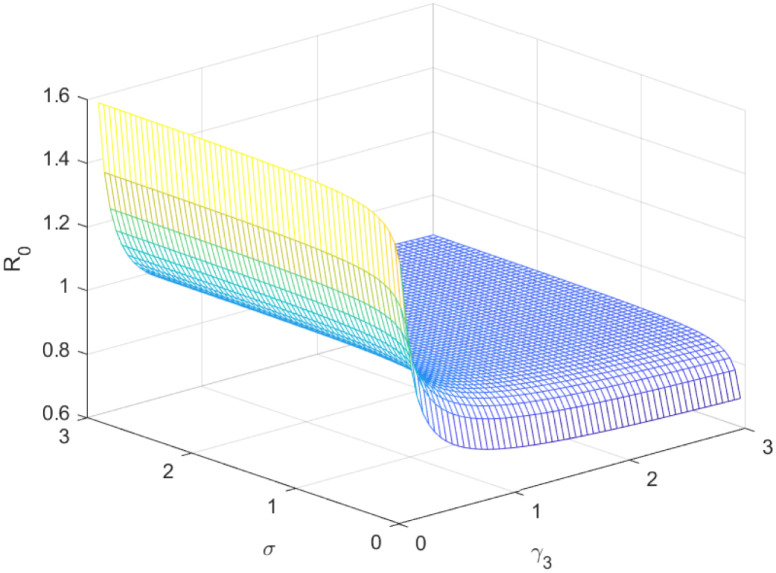
Effect of *σ* and *γ*_3_ on reproductive number.

**Fig 8:**
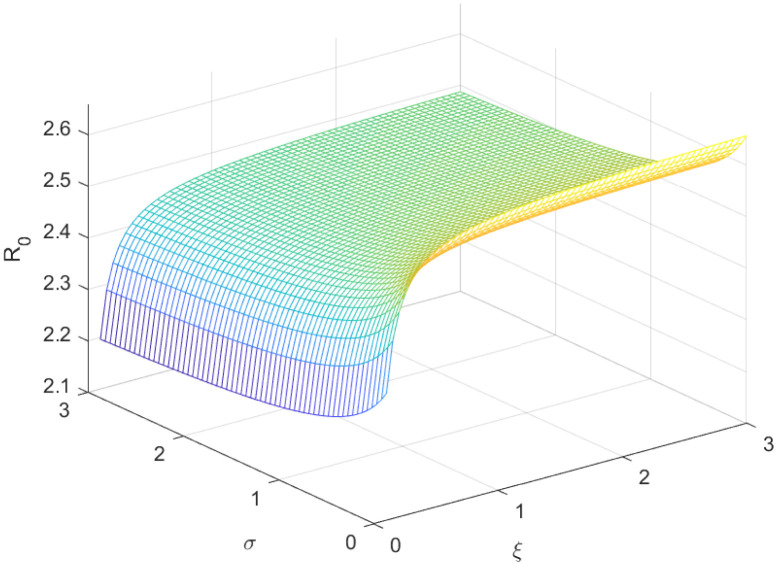
Effect of *σ* and *ξ* on reproductive number.

**Fig 9:**
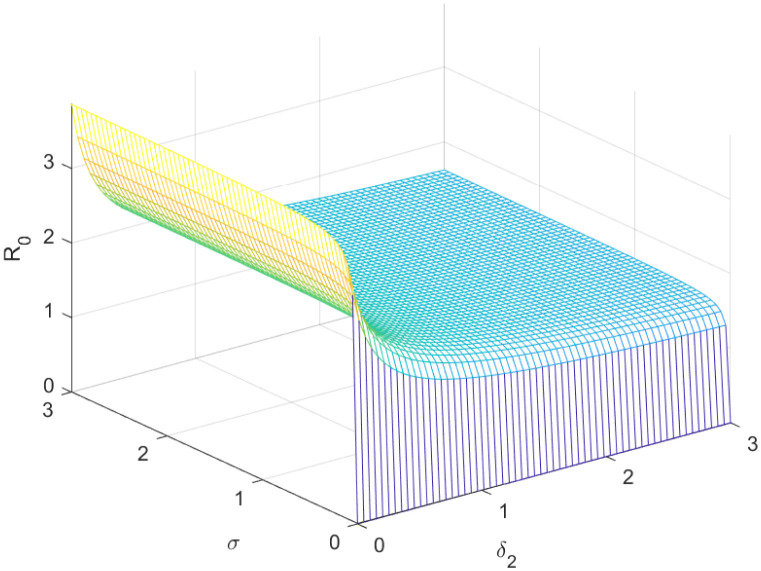
Effect of *σ* and *δ*_2_ on reproductive number.

**Fig 10:**
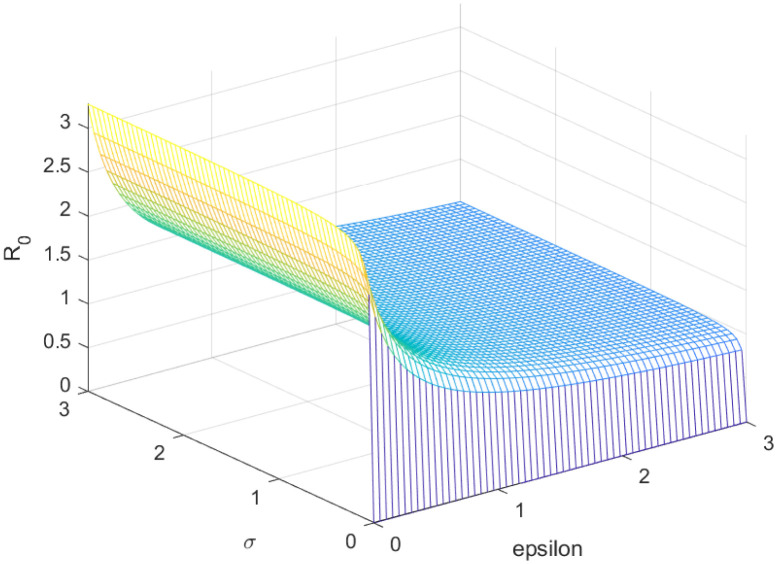
Effect of *σ* and *ϵ* on reproductive number.

**Fig 11:**
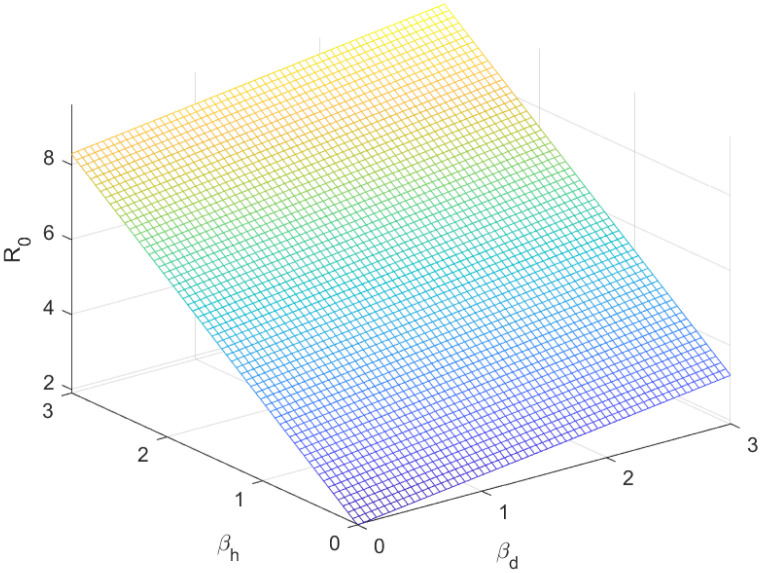
Effect of *β*_*h*_ and *β*_*d*_ on reproductive number.

**Fig 12:**
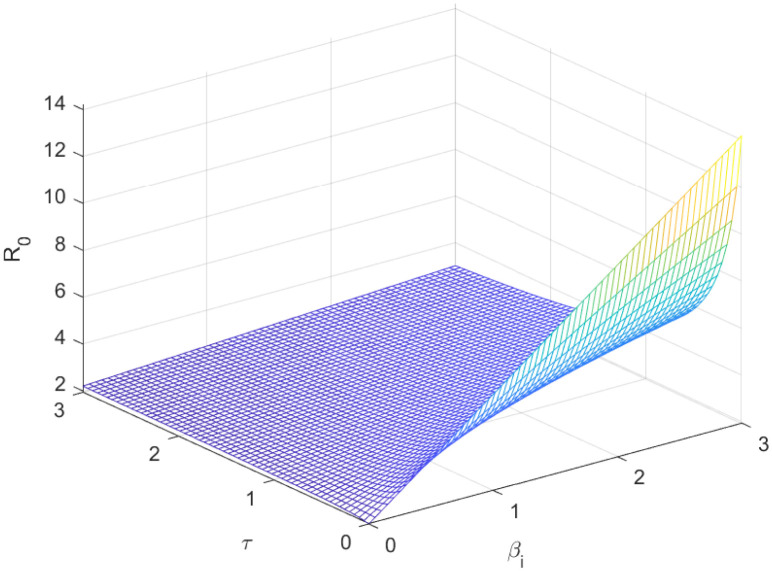
Effect of *τ* and *β*_*i*_ on reproductive number.

**Fig 13:**
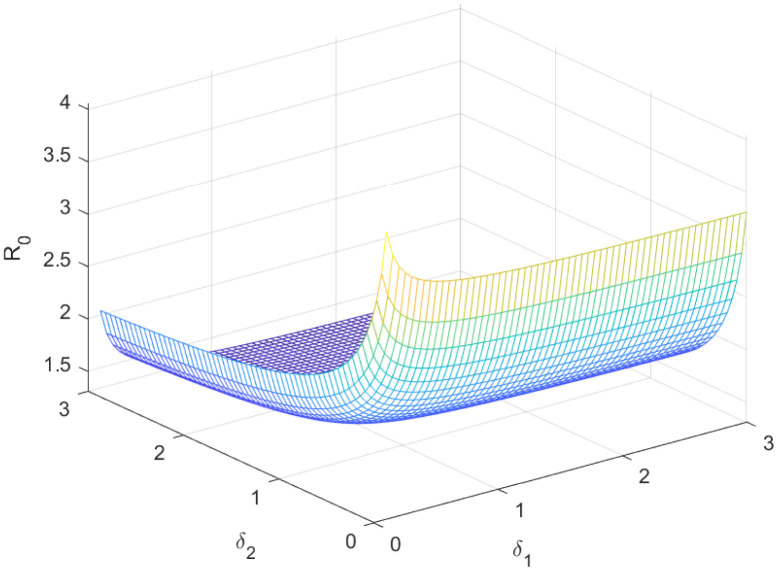
Effect of *δ*_2_ and *δ*_1_ on reproductive number.

**Fig 14:**
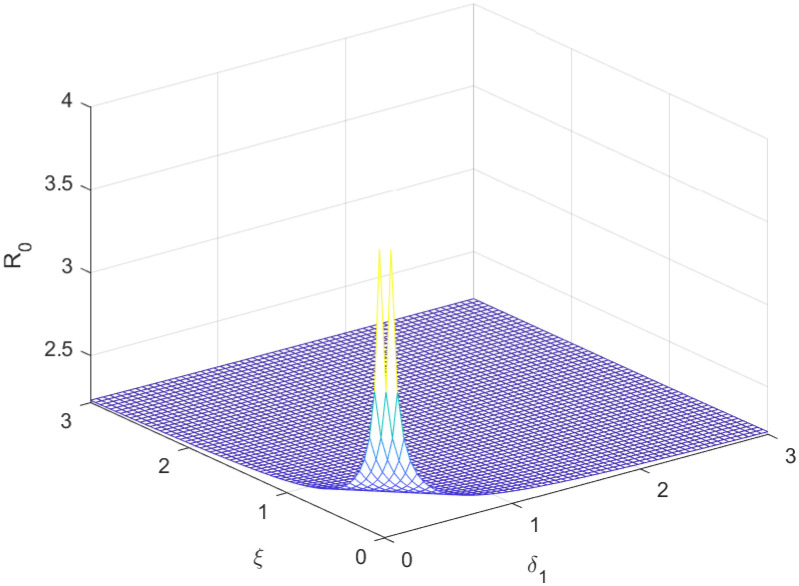
Effect of *ξ* and *δ*_1_ on reproductive number.

**Fig 15:**
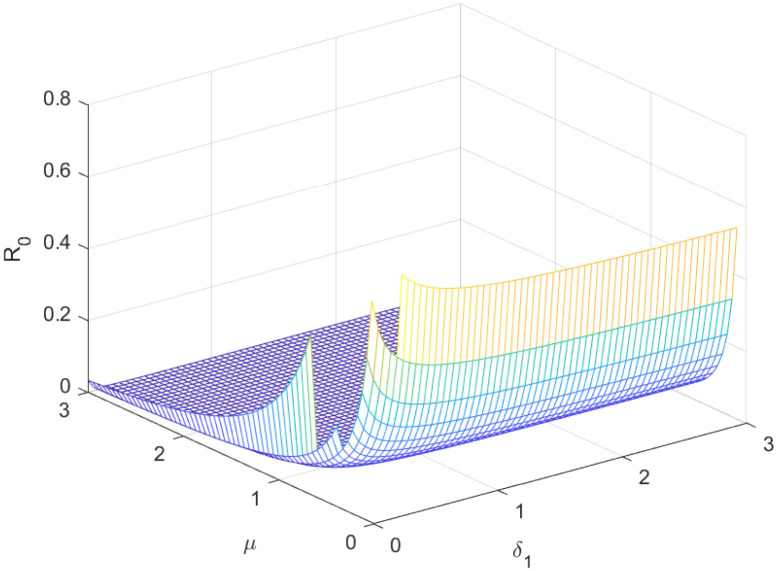
Effect of *μ* and *δ*_1_ on reproductive number.

**Fig 16:**
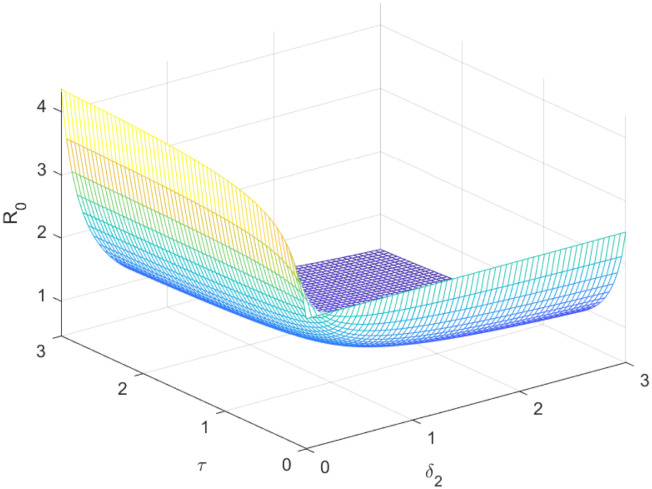
Effect of *τ* and *δ*_2_ on reproductive number.

The reproductive number serves as a crucial epidemiological metric for evaluating the transmission potential of an infectious disease within a susceptible population. It quantifies the average number of secondary infections generated by a single infected individual in a population that is entirely susceptible to the disease. As is common knowledge, if the fundamental reproduction number *R*_0_ < 1, the infection will ultimately come to an end. However, the illness will spread across the population if *R*_0_ > 1. Figs 2 to 16 illustrate the influence of various parameters on the reproductive number providing insights into the biological feasibility and disease control.

### 3.3 Sensitivity analysis

Sensitivity analysis is employed to investigate how the parameters impact the proposed Ebola virus model. Finding the parameters that are most likely to be affected by a slight change in numeric value is very important. The reproductive number *R*_0_ is:

$$\begin{array}{c} R_{0}=\frac{\sigma }{q_{1}q_{2}q_{3}q_{4}q_{5}}[q_{3}\left(\beta_{i}\left(\gamma_{3}q_{4}+\mu \left(\gamma_{2}+\delta_{2}\right)+\mu^{2}\right)+\beta_{r}\left(\gamma_{1}q_{4}+\gamma_{2}\tau \right)+\beta_{h}\tau q_{2}\right)+\beta_{d}\varepsilon (\left(\gamma_{3}q_{4}+\mu \left(\gamma_{2}+\delta_{2}\right)\right)+\mu^{2}]. \end{array}$$ (11)

The sensitivity of *R*_0_ may be investigated by taking into account given the relevant factors, partial derivatives of the reproductive number.

$$\begin{array}{cc} \frac{\partial R_{0}}{\partial \sigma } & =\frac{\rho }{\left(\gamma_{3}+\mu \right)\left(\mu +\sigma \right)\left(\delta_{2}+\xi \right)\left(\gamma_{2}+\mu +\delta_{2}\right)\left(\gamma_{1}+\mu +\tau +\varepsilon \right)}\\ & -\frac{\sigma \rho }{\left(\gamma_{3}+\mu \right){\left(\mu +\sigma \right)}^{2}\left(\delta_{2}+\xi \right)\left(\gamma_{2}+\mu +\delta_{2}\right)\left(\gamma_{1}+\mu +\tau +\varepsilon \right)}>0, \end{array}$$

$$\begin{array}{cc} \frac{\partial R_{0}}{\partial \mu } & =\frac{\sigma \xi (2\mu +\beta_{r}\gamma_{1}+\beta_{i}\left(\gamma_{2}+\gamma_{3}+2\mu +\delta_{2}\right)+\beta_{h}\tau +\beta_{d}\varepsilon \left(\gamma_{2}+\gamma_{3}+\delta_{2}\right))}{\left(\gamma_{3}+\mu \right)\left(\mu +\sigma \right)\left(\delta_{2}+\zeta \right)\left(\gamma_{2}+\mu +\delta_{2}\right)\left(\gamma_{1}+\mu +\tau +\varepsilon \right)}\\ & -\frac{\sigma \rho }{\left(\gamma_{3}+\mu \right)\left(\mu +\sigma \right)\left(\delta_{2}+\xi \right)\left(\gamma_{2}+\mu +\delta_{2}\right){\left(\gamma_{1}+\mu +\tau +\upsilon \right)}^{2}}\\ & -\frac{\sigma \rho }{{\left(\gamma_{3}+\mu \right)}^{2}\left(\mu +\sigma \right)\left(\delta_{2}+\xi \right)\left(\gamma_{2}+\mu +\delta_{2}\right)\left(\gamma_{1}+\mu +\tau +\upsilon \right)}\\ & -\frac{\sigma \rho }{\left(\gamma_{3}+\mu \right){\left(\mu +\sigma \right)}^{2}\left(\delta_{2}+\xi \right)\left(\gamma_{2}+\mu +\delta_{2}\right)\left(\gamma_{1}+\mu +\tau +\upsilon \right)}\\ & -\frac{\sigma \rho }{\left(\gamma_{3}+\mu \right)\left(\mu +\sigma \right)\left(\delta_{2}+\xi \right)\left(\gamma_{2}+\mu +\delta_{2}\right){\left(\gamma_{1}+\mu +\tau +\varepsilon \right)}^{2}}<0, \end{array}$$

$$\frac{\partial R_{0}}{\partial \beta_{i}}=\frac{\sigma \xi (\mu \left(\gamma_{2}+\delta_{2}\right)+\gamma_{3}\left(\gamma_{2}+\mu +\delta_{2}\right)+\mu^{2})}{\left(\gamma_{3}+\mu \right)\left(\mu +\sigma \right)\left(\delta_{2}+\xi \right)\left(\gamma_{2}+\mu +\delta_{2}\right)\left(\gamma_{1}+\mu +\tau +\varepsilon \right)}>0,$$

$$\frac{\partial R_{0}}{\partial \beta_{d}}=\frac{\sigma \varepsilon \xi (\mu \left(\gamma_{2}+\delta_{2}\right)+\gamma_{3}\left(\gamma_{2}+\mu +\delta_{2}\right))}{\left(\gamma_{3}+\mu \right)\left(\mu +\sigma \right)\left(\delta_{2}+\xi \right)\left(\gamma_{2}+\mu +\delta_{2}\right)\left(\gamma_{1}+\mu +\tau +\varepsilon \right)}>0,$$

$$\frac{\partial R_{0}}{\partial \beta_{h}}=\frac{\sigma \tau \xi }{\left(\mu +\sigma \right)\left(\delta_{2}+\xi \right)\left(\gamma_{2}+\mu +\delta_{2}\right)\left(\gamma_{1}+\mu +\tau +\varepsilon \right)}>0,$$

$$\frac{\partial R_{0}}{\partial \beta_{r}}=\frac{\sigma \xi (\gamma_{2}\tau +\gamma_{1}\left(\gamma_{2}+\mu +\delta_{2}\right))}{\left(\gamma_{3}+\mu \right)\left(\mu +\sigma \right)\left(\delta_{2}+\xi \right)\left(\gamma_{2}+\mu +\delta_{2}\right)\left(\gamma_{1}+\mu +\tau +\varepsilon \right)}>0,$$

$$\begin{array}{cc} \frac{\partial R_{0}}{\partial \gamma_{1}} & =\frac{\beta_{r}\sigma \xi }{\left(\gamma_{3}+\mu \right)\left(\mu +\sigma \right)\left(\delta_{2}+\xi \right)\left(\gamma_{1}+\mu +\tau +\varepsilon \right)}\\ & -\frac{\sigma \rho }{\left(\gamma_{3}+\mu \right)\left(\mu +\sigma \right)\left(\delta_{2}+\xi \right)\left(\gamma_{2}+\mu +\delta_{2}\right){\left(\gamma_{1}+\mu +\tau +\varepsilon \right)}^{2}}<0, \end{array}$$

$$\begin{array}{cc} \frac{\partial R_{0}}{\partial \varepsilon } & =\frac{\beta_{d}\sigma \xi \left(\mu \left(\gamma_{2}+\delta_{2}\right)+\gamma_{3}\left(\gamma_{2}+\mu +\delta_{2}\right)\right)}{\left(\gamma_{3}+\mu \right)\left(\mu +\sigma \right)\left(\delta_{2}+\xi \right)\left(\gamma_{2}+\mu +\delta_{2}\right)\left(\gamma_{1}+\mu +\tau +\varepsilon \right)}\\ & -\frac{\sigma \rho }{\left(\gamma_{3}+\mu \right)\left(\mu +\sigma \right)\left(\delta_{2}+\xi \right)\left(\gamma_{2}+\mu +\delta_{2}\right){\left(\gamma_{1}+\mu +\tau +\varepsilon \right)}^{2}}<0, \end{array}$$

$$\frac{\partial R_{0}}{\partial \delta_{1}}=\frac{\sigma }{\left(\gamma_{3}+\mu \right)\left(\mu +\sigma \right)\left(\delta_{2}+\xi \right)\left(\gamma_{2}+\mu +\delta_{2}\right)\left(\gamma_{1}+\mu +\tau +\varepsilon \right)}>0,$$

$$\begin{array}{cc} \frac{\partial R_{0}}{\partial \delta_{2}} & =\frac{\sigma \xi (\beta_{r}\gamma_{1}+\beta_{i}\left(\gamma_{3}+\mu \right)+\beta_{d}\varepsilon \left(\gamma_{3}+\mu \right))}{\left(\gamma_{3}+\mu \right)\left(\mu +\sigma \right)\left(\delta_{2}+\xi \right)\left(\gamma_{2}+\mu +\delta_{2}\right)\left(\gamma_{1}+\mu +\tau +\varepsilon \right)}\\ & -\frac{\sigma \rho }{\left(\gamma_{3}+\mu \right)\left(\mu +\sigma \right){\left(\delta_{2}+\xi \right)}^{2}\left(\gamma_{2}+\mu +\delta_{2}\right)\left(\gamma_{1}+\mu +\tau +\varepsilon \right)}\\ & -\frac{\sigma \rho }{\left(\gamma_{3}+\mu \right)\left(\mu +\sigma \right)\left(\delta_{2}+\xi \right){\left(\gamma_{2}+\mu +\delta_{2}\right)}^{2}\left(\gamma_{1}+\mu +\tau +\varepsilon \right)}<0, \end{array}$$

$$\begin{array}{cc} \frac{\partial R_{0}}{\partial \gamma_{2}} & =\frac{\sigma \xi (\beta_{i}\left(\gamma_{3}+\mu \right)+\beta_{r}\left(\gamma_{1}+\tau \right)+\beta_{d}\varepsilon \left(\gamma_{3}+\mu \right))}{\left(\gamma_{3}+\mu \right)\left(\mu +\sigma \right)\left(\delta_{2}+\xi \right)\left(\gamma_{2}+\mu +\delta_{2}\right)\left(\gamma_{1}+\mu +\tau +\varepsilon \right)}\\ & -\frac{\sigma \rho }{\left(\gamma_{3}+\mu \right)\left(\mu +\sigma \right)\left(\delta_{2}+\xi \right){\left(\gamma_{2}+\mu +\delta_{2}\right)}^{2}\left(\gamma_{1}+\mu +\tau +\varepsilon \right)}<0, \end{array}$$

$$\begin{array}{cc} \frac{\partial R_{0}}{\partial \tau } & =\frac{\sigma \xi (\beta_{r}\gamma_{2}+\beta_{h}\left(\gamma_{3}+\mu \right))}{\left(\gamma_{3}+\mu \right)\left(\mu +\sigma \right)\left(\delta_{2}+\xi \right)\left(\gamma_{2}+\mu +\delta_{2}\right)\left(\gamma_{1}+\mu +\tau +\varepsilon \right)}\\ & -\frac{\sigma \rho }{\left(\gamma_{3}+\mu \right)\left(\mu +\sigma \right)\left(\delta_{2}+\xi \right)\left(\gamma_{2}+\mu +\delta_{2}\right){\left(\gamma_{1}+\mu +\tau +\varepsilon \right)}^{2}}<0, \end{array}$$

$$\begin{array}{cc} \frac{\partial R_{0}}{\partial \gamma_{3}} & =\frac{\sigma \xi (\beta_{h}\tau +\beta_{i}\left(\gamma_{2}+\mu +\delta_{2}\right)+\beta_{d}\varepsilon \left(\gamma_{2}+\mu +\delta_{2}\right))}{{\left(\gamma_{3}+\mu \right)}^{2}\left(\mu +\sigma \right)\left(\delta_{2}+\xi \right)\left(\gamma_{2}+\mu +\delta_{2}\right)\left(\gamma_{1}+\mu +\tau +\varepsilon \right)}\\ & -\frac{\sigma \rho }{{\left(\gamma_{3}+\mu \right)}^{2}\left(\mu +\sigma \right)\left(\delta_{2}+\xi \right)\left(\gamma_{2}+\mu +\delta_{2}\right)\left(\gamma_{1}+\mu +\tau +\varepsilon \right)}<0, \end{array}$$

$$\begin{array}{cc} \frac{\partial R_{0}}{\partial \xi } & =\frac{\sigma \rho_{1}}{\left(\gamma_{3}+\mu \right)\left(\mu +\sigma \right)\left(\delta_{2}+\xi \right)\left(\gamma_{2}+\mu +\sigma^{2}\right)\left(\gamma_{1}+\mu +\tau +\varepsilon \right)}\\ & -\frac{\sigma \rho }{\left(\gamma_{3}+\mu \right)\left(\mu +\sigma \right){\left(\delta_{2}+\xi \right)}^{2}\left(\gamma_{2}+\mu +\delta_{2}\right)\left(\gamma_{1}+\mu +\tau +\varepsilon \right)}<0, \end{array}$$

where

$$\begin{array}{cc} \rho & =\delta_{1}+\xi (\beta_{i}\left(\mu \left(\gamma_{2}+\delta_{2}\right)+\gamma_{3}\left(\gamma_{2}+\mu +\delta_{2}\right)+\mu^{2}\right)+\beta_{r}\left(\gamma_{2}\tau +\gamma_{1}\left(\gamma_{2}+\mu +\delta_{2}\right)\right)\\ & +\mu^{2}+\beta_{d}\varepsilon \left(\mu \left(\gamma_{2}+\delta_{2}\right)+\gamma_{3}\left(\gamma_{2}+\mu +\delta_{2}\right)\right)+\beta_{h}\tau \left(\gamma_{3}+\mu \right)) \end{array}$$

$$\begin{array}{c} \rho_{1}=\beta_{i}\left(\mu \left(\gamma_{2}+\delta_{2}\right)+\gamma_{3}\left(\gamma_{2}+\mu +\delta_{2}\right)+\mu^{2}\right)+\beta_{r}\left(\gamma_{2}\tau +\gamma_{1}\left(\gamma_{2}+\mu +\sigma^{2}\right)\right)\\ +\mu^{2}+\beta_{d}\varepsilon \left(\mu \left(\gamma_{2}+\delta_{2}\right)+\gamma_{3}\left(\gamma_{2}+\mu +\delta_{2}\right)\right)+\beta_{h}\tau \left(\gamma_{3}+\mu \right) \end{array}$$

sensitivity analysis plays a crucial role in understanding how variations in different parameters influence *R*_0_ and helps in devising more effective strategies for disease control and prevention. Positive sensitivity indices increase *R*_0_, and negative indices decrease it according to values given in Table 1 and shown in Fig 17, highlighting key aspects influencing transmission potential, allowing for the identification of key variables and their effects on the spread of the Ebola virus.

**Fig 17:**
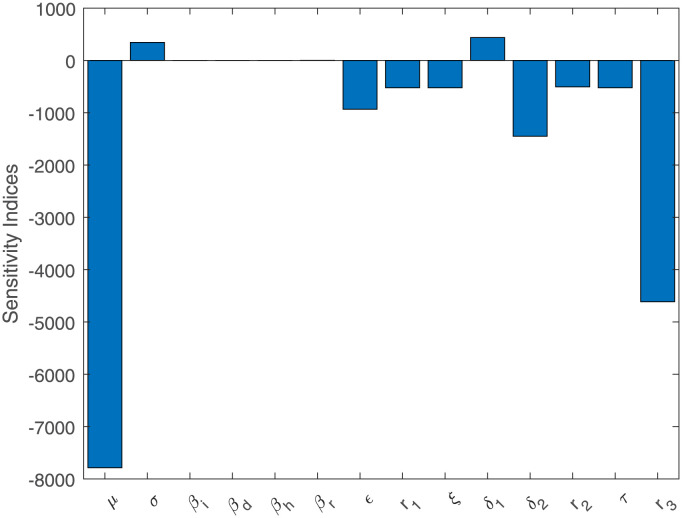
Results from the PRCC for the relevance of the variables in *R*_0_.

## 4 Stability analysis of the proposed model

The proposed model system (7) undergoes qualitative analysis to gain insights into its dynamical properties thereby improving our understanding of how control methods influence the dynamics of infectious disease transmission. Initially, the stability properties of the infectious model are investigated.

### 4.1 Local stability analysis

**Theorem 4.1** A Ebola Virus free equilibrium *E*_0_ is asymptotic locally stable when *R*_0_ < 0. Instability exists if *R*_0_ > 0.

**Proof:** The Jacobian J for the system (7) at *E*_0_ can be written as

$$J\left(E_{0}\right)=[\begin{array}{cccccccc} -\mu & 0 & -\beta_{i} & -\beta_{r} & -\beta_{d} & -\beta_{h} & 0 & 0\\ 0 & -\mu -\sigma & \beta_{i} & \beta_{r} & \beta_{d} & \beta_{h} & 0 & 0\\ 0 & \sigma & -\varepsilon -\gamma_{1}-\mu -\tau & 0 & 0 & 0 & 0 & 0\\ 0 & 0 & \gamma_{1} & -\gamma_{3}-\mu & 0 & \gamma_{2} & 0 & 0\\ 0 & 0 & \varepsilon & 0 & -\delta_{1}-\xi & 0 & 0 & 0\\ 0 & 0 & \tau & 0 & 0 & -\gamma_{2}-\mu -\delta_{2} & 0 & 0\\ 0 & 0 & 0 & 0 & \delta_{1} & \delta_{2} & -\xi & 0\\ 0 & 0 & 0 & \gamma_{3} & 0 & 0 & 0 & -\mu \end{array}]$$

For the equilibrium point *E*_0_ to be locally asymptotically stable, it is necessary and sufficient to confirm that all the eigenvalues of the matrix *J*(*E*_0_) satisfy the stability condition:

$$\begin{array}{c} |\text{arg}(\lambda_{i})|>\frac{\eta \pi }{2} \end{array}$$ (12)

When substituting the parameter values from Table 1 into the Jacobian matrix the resulting eigenvalues are as follows: λ_1_ = −0.0140, λ_2_ = −0.0140, λ_3_ = −0.0140, λ_4_ = −0.0392, λ_5_ = −0.1286, λ_6_ = −0.4739, λ_7_ = −0.4739, λ_8_ = −0.4739. It can be concluded that all eigenvalues satisfy the stability condition (12). The point *E*_0_ is locally asymptotically stable if *R*_0_ < 1.

### 4.2 Global stability analysis

#### 4.2.1 First derivative of Lyapunov

In the case of the endemic Lyapunov function, {*S*, *E*, *I*, *R*, *D*, *H*, *B*, *C*} D0PCtηL < 0 is the endemic equilibrium E*.

**Theorem 4.2** The endemic equilibrium points E* of the Ebola virus model exhibit global asymptotic stability when the reproductive number *R*_0_ > 1.

**Proof:** The Lyapunov function can be formulated as:

$$\begin{array}{cc} L(S^{*},E^{*},I^{*},R^{*},D^{*},H^{*},B^{*},C^{*}) & =(S-S^{*}-S^{*}\;\text{log}\;\frac{S^{*}}{S})+(E-E^{*}-E^{*}\;\text{log}\;\frac{E^{*}}{E})\\ & +(I-I^{*}-I^{*}\;\text{log}\;\frac{I^{*}}{I})+(R-R^{*}-R^{*}\;\text{log}\;\frac{R^{*}}{R})\\ & +(D-D^{*}-D^{*}\;\text{log}\;\frac{D^{*}}{D})+(H-H^{*}-H^{*}\;\text{log}\;\frac{H^{*}}{H})\\ & +(B-B^{*}-B^{*}\;\text{log}\;\frac{B^{*}}{B})+(C-C^{*}-C^{*}\;\text{log}\;\frac{C^{*}}{C}) \end{array}$$ (13)

Consequently, by taking the derivative with respect to t on both sides, we obtain:

D0PCtηL≤(S-S*S)D0PCtηS+(E-E*E)D0PCtηE+(I-I*I)D0PCtηI+(R-R*R)D0PCtηR+(D-D*D)D0PCtηD+(H-H*H)D0PCtηH+(B-B*B)D0PCtηB+(C-C*C)D0PCtηC (14)

Now, we may express their values for derivatives as follows

D0PCtηL≤(S-S*S)(μN-βiNSI-βhNSH-βdNSD-βrNSR-μS)+(E-E*E)(βiNSI+βhNSH+βdNSD+βrNSR-σE-μE)+(I-I*I)(σE-(γ1+ε+τ+μ)I)+(R-R*R)(γ1I-γ2H-(γ3+μ)R)+(D-D*D)(εI-δ1D-ξD)+(H-H*H)(τI-(γ2+δ2+μ)H)+(B-B*B)(δ1D+δ2H-ξB)+(C-C*C)(γ3R-μC) (15)

Putting *S* = *S* − S*, *E* = *E* − E*, *S* = *I* − I*, *R* = *R* − R*, *D* = *D* − D*, *H* = *H* − H*, *B* = *B* − B* and *C* = *C* − C* leads to.

D0PCtηL≤(S-S*S)(μN-βiN(S-S*)(I-I*)-βhN(S-S*)(H-H*)-βdN(S-S*)(D-D*)-βrN(S-S*)(R-R*)-μ(S-S*))+(E-E*E)(βiN(S-S*)(I-I*)+βhN(S-S*)(H-H*)+βdN(S-S*)(D-D*)+βrN(S-S*)(R-R*)-σ(E-E*)-μ(E-E*))+(I-I*I)(σ(E-E*)-(γ1+ε+τ+μ)(I-I*))+(R-R*R)(γ1(I-I*)-γ2(H-H*)-(γ3+μ)(R-R*))+(D-D*D)(ε(I-I*)-δ1(D-D*)-ξ(D-D*))+(H-H*H)(τ(I-I*)-(γ2+δ2+μ)(H-H*))+(B-B*B)(δ1(D-D*)+δ2(H-H*)-ξ(B-B*))+(C-C*C)(γ3R-μ(C-C*))

After some calculations, to avoid the complexity we write

D0PCtηL≤θ-ϕ

$$\begin{array}{cc} \theta & =\mu N+\frac{{\left(S-S^{*}\right)}^{2}}{S}\frac{\beta_{i}I^{*}}{N}+\frac{{\left(S-S^{*}\right)}^{2}}{S}\frac{\beta_{h}H^{*}}{N}+\frac{{\left(S-S^{*}\right)}^{2}}{S}\frac{\beta_{d}D^{*}}{N}+\frac{{\left(S-S^{*}\right)}^{2}}{S}\frac{\beta_{r}R^{*}}{N}+\frac{\beta_{i}}{N}SI+\frac{\beta_{i}}{N}S^{*}I^{*}\\ & +\frac{E^{*}}{E}\frac{\beta_{i}}{N}SI^{*}+\frac{E^{*}}{E}\frac{\beta_{i}}{N}S^{*}I+\frac{\beta_{h}}{N}SH+\frac{\beta_{h}}{N}S^{*}H^{*}+\frac{E^{*}}{E}\frac{\beta_{h}}{N}SH^{*}+\frac{E^{*}}{E}\frac{\beta_{h}}{N}S^{*}H+\frac{\beta_{d}}{N}SD+\frac{\beta_{d}}{N}S^{*}D^{*}\\ & +\frac{E^{*}}{E}\frac{\beta_{d}}{N}SD^{*}+\frac{E^{*}}{E}\frac{\beta_{d}}{N}S^{*}D+\frac{\beta_{r}}{N}SR+\frac{\beta_{r}}{N}S^{*}R^{*}+\frac{E^{*}}{E}\frac{\beta_{r}}{N}SR^{*}+\frac{E^{*}}{E}\frac{\beta_{r}}{N}S^{*}R+\sigma E+\frac{I^{*}}{I}\sigma E^{*}+\gamma_{1}I+\frac{R^{*}}{R}\gamma_{1}I^{*}\\ & +\gamma_{2}H+\frac{R^{*}}{R}\gamma_{2}H^{*}+\varepsilon I+\frac{D^{*}}{D}\varepsilon I^{*}+\tau I+\frac{H^{*}}{H}\tau I^{*}+\delta_{1}D+\frac{B^{*}}{B}\delta_{1}D^{*}+\delta_{2}H+\frac{B^{*}}{B}\delta_{2}H^{*}+\gamma_{3}R+\frac{C^{*}}{C}\gamma_{3}R^{*} \end{array}$$

$$\begin{array}{cc} \phi & =\frac{S^{*}}{S}\mu N+\frac{\beta_{i}}{N}\frac{{\left(S-S^{*}\right)}^{2}}{S}I+\frac{\beta_{h}}{N}\frac{{\left(S-S^{*}\right)}^{2}}{S}H+\frac{\beta_{d}}{N}\frac{{\left(S-S^{*}\right)}^{2}}{S}D+\frac{\beta_{r}}{N}\frac{{\left(S-S^{*}\right)}^{2}}{S}R+\mu \frac{{\left(S-S^{*}\right)}^{2}}{S}+\frac{\beta_{i}}{N}S^{*}I\\ & +\frac{\beta_{i}}{N}SI^{*}+\frac{E^{*}}{E}\frac{\beta_{i}}{N}SI+\frac{E^{*}}{E}\frac{\beta_{i}}{N}S^{*}I^{*}+\frac{\beta_{h}}{N}S^{*}H+\frac{\beta_{h}}{N}SH^{*}+\frac{E^{*}}{E}\frac{\beta_{h}}{N}SH+\frac{E^{*}}{E}\frac{\beta_{h}}{N}S^{*}H^{*}+\frac{\beta_{d}}{N}SD^{*}+\frac{\beta_{d}}{N}S^{*}D\\ & +\frac{E^{*}}{E}\frac{\beta_{d}}{N}SD+\frac{E^{*}}{E}\frac{\beta_{d}}{N}S^{*}D^{*}+\frac{\beta_{r}}{N}SR^{*}+\frac{\beta_{r}}{N}S^{*}R+\frac{E^{*}}{E}\frac{\beta_{r}}{N}SR+\frac{E^{*}}{E}\frac{\beta_{r}}{N}S^{*}R^{*}+\sigma \frac{{\left(E-E^{*}\right)}^{2}}{E}+\mu \frac{{\left(E-E^{*}\right)}^{2}}{E}\\ & +\sigma E^{*}+\frac{I^{*}}{I}\sigma E+\gamma_{1}\frac{{\left(I-I\right)}^{2}}{I}+\varepsilon \frac{{\left(I-I\right)}^{2}}{I}+\tau \frac{{\left(I-I\right)}^{2}}{I}+\mu \frac{{\left(I-I\right)}^{2}}{I}+\gamma_{1}I^{*}+\frac{R^{*}}{R}\gamma_{1}I\\ & +\gamma_{2}H^{*}+\frac{R^{*}}{R}\gamma_{2}H+\left(\gamma_{3}+\mu \right)\frac{{\left(R-R^{*}\right)}^{2}}{R}-\varepsilon I^{*}+\frac{D^{*}}{D}\varepsilon I\\ & +\delta_{1}\frac{{\left(D-D^{*}\right)}^{2}}{D}+\xi \frac{{\left(D-D^{*}\right)}^{2}}{D}+\tau I^{*}+\frac{H^{*}}{H}\tau I+(\gamma_{2}+\delta_{2}+\mu )\frac{{\left(H-H^{*}\right)}^{2}}{H}\\ & +\delta_{1}D^{*}+\frac{B^{*}}{B}\delta_{1}D+\delta_{2}H^{*}+\frac{B^{*}}{B}\delta_{2}H+\xi \frac{{\left(B-B^{*}\right)}^{2}}{B}+\gamma_{3}R^{*}\\ & +\frac{C^{*}}{C}\gamma_{3}R+\mu \frac{{\left(C-C^{*}\right)}^{2}}{C} \end{array}$$

It is achieved that if *θ* < *ϕ*, this yields D0PCtηL<0, however when *S* = S*, *E* = E*, *I* = I*, *R* = R*, *D* = D*, *H* = H*, *B* = B*andC = C*.

0=θ-ϕ⇒D0PCtηL=0. (16)

The existence of the largest compact invariant set in the proposed model can be established.

$$\begin{array}{c} \{(S^{*},E^{*},I^{*},R^{*},D^{*},H^{*},B^{*},C_{h}^{*})\}. \end{array}$$ (17)

Utilizing Lasalle’s invariance concept, it can be deduced that E* is globally asymptotically stable within the region if the *θ* < *ϕ*.

#### 4.2.2 Second derivative of Lyapunov

Epidemiology is one of the disciplines that frequently uses Lyapunov functions. They are used to assess stability. Though the sign of the first derivative can suggest stability, it may not always be possible to tell whether it is a local maximum or minimum. As a result, it is recommended to investigate the second derivative. In this section, our focus is on examining the second derivative of the Lyapunov function for our model.

D˙0PCtηL≤{1−S*SD0PCtηS+1−E*ED0PCtηE+1−I*ID0PCtηI+1−R*RD0PCtηR+1−D*DD0PCtηD+1−H*HD0PCtηH+1−B*BD0PCtηB+1−C*CD0PCtηC}=D0PCtηSS2S*+D0PCtηEE2+E*D0PCtηII2+I*D0PCtηRR2R*+D0PCtηDD2D*+D0PCtηHH2+H*D0PCtηBB2+B*D0PCtηCC2C*+1−S*SD˙0PCtηS+1−E*ED˙0PCtβE+1−I*ID˙0PCtηI+1−R*RD˙0PCtηR+1−D*DD˙0PCtηD+1−H*HD˙0PCtηH+1−B*BD˙0PCtηB+1−C*CD˙0PCtηC (18)

Here

D˙0PCtηS=-βiN((D0PCtηS)I+S(D0PCtηI))-βhN((D0PCtηS)H+S(D0PCtηH))-βdN((D0PCtηS)D+S(D0PCtηD))-βrN((D0PCtηS)R+S(D0PCtηR))-μ(D0PCtηS)D˙0PCtηE=βiN((D0PCtηS)I+S(D0PCtηI))+βhN((D0PCtηS)H+S(D0PCtηH))+βdN((D0PCtηS)D+S(D0PCtηD))+βrN((D0PCtηS)R+S(D0PCtηR))-σ(D0PCtηE)-μ(D0PCtηE)D˙0PCtηI=σ(D0PCtηE)-(γ1+ε+τ+μ)(D0PCtηI)D˙0PCtηR=γ1(D0PCtηI)-γ2(D0PCtηH)-(γ3+μ)(D0PCtηR)D˙0PCtηD=ε(D0PCtηI)-(δ1+ξ)(D0PCtηD)D˙0PCtηH=τ(D0PCtηI)-(γ2+δ2+μ)(D0PCtηH)D˙0PCtηB=δ1(D0PCtηD)+δ2(D0PCtηH)-ξ(D0PCtηB)D˙0PCtηC=γ3(D0PCtηR)-μ(D0PCtηC) (19)

Then we have

=(D0PCtηSS)2S*+(D0PCtηEE)2E*+(D0PCtηII)2I*+(D0PCtηRR)2R*+(D0PCtηDD)2D*+(D0PCtηHH)2H*+(D0PCtηBB)2B*+(D0PCtηCC)2C*+(1-S*S)(-βiN((D0PCtηS)I+S(D0PCtηI))-βhN((D0PCtηS)H+S(D0PCtηH))-βdN((D0PCtηS)D+S(D0PCtηD))-βrN((D0PCtηS)R+S(D0PCtηR))-μ(D0PCtηS))+(1-E*E)(βiN((D0PCtηS)I+S(D0PCtηI))+βhN((D0PCtηS)H+S(D0PCtηH))+βdN((D0PCtηS)D+S(D0PCtηD))+βrN((D0PCtηS)R+S(D0PCtηR))-σ(D0PCtηE)-μ(D0PCtηE))+(1-I*I)(σ(D0PCtηE)-(γ1+ε+τ+μ)(D0PCtηI))+(1-R*R)(γ1(D0PCtηI)-γ2(D0PCtηH)-(γ3+μ)(D0PCtηR))+(1-D*D)(ε(D0PCtηI)-(δ1+ξ)(D0PCtηD))+(1-H*H)(τ(D0PCtηI)-(γ2+δ2+μ)(D0PCtηH))+(1-B*B)(δ1(D0PCtηD)+δ2(D0PCtηH)-ξ(D0PCtηB))+(1-C*C)(γ3(D0PCtηR)-μ(D0PCtηC)) (20)

And

=(D0PCtηSS)2S*+(D0PCtηEE)2E*+(D0PCtηII)2I*+(D0PCtηRR)2R*+(D0PCtηDD)2D*+(D0PCtηHH)2H*+(D0PCtηBB)2B*+(D0PCtηCC)2C*+(1-S*S)D˙0PCtηS+(1-E*E)D˙0PCtηE+(1-I*I)D˙0PCtηI+(1-R*R)D˙0PCtηR+(1-D*D)D˙0PCtηD+(1-H*H)D˙0PCtηH+(1-B*B)D˙0PCtηB+(1-C*C)D˙0PCtηC=Π˙(S,E,I,R,D,H,B,C)+(1-S*S)(-βiN((D0PCtηS)I+S(D0PCtηI))-βhN((D0PCtηS)H+S(D0PCtηH))-βdN((D0PCtηS)D+S(D0PCtηD))-βrN((D0PCtηS)R+S(D0PCtηR))-μ(D0PCtηS))+(1-E*E)(βiN((D0PCtηS)I+S(D0PCtηI))+βhN((D0PCtηS)H+S(D0PCtηH))+βdN((D0PCtηS)D+S(D0PCtηD))+βrN((D0PCtηS)R+S(D0PCtηR))-σ(D0PCtηE)-μ(D0PCtηE))+(1-I*I)(σ(D0PCtηE)-(γ1+ε+τ+μ)(D0PCtηI))+(1-R*R)(γ1(D0PCtηI)-γ2(D0PCtηH)-(γ3+μ)(D0PCtηR))+(1-D*D)(ε(D0PCtηI)-(δ1+ξ)(D0PCtηD))+(1-H*H)(τ(D0PCtηI)-(γ2+δ2+μ)(D0PCtηH))+(1-B*B)(δ1(D0PCtηD)+δ2(D0PCtηH)-ξ(D0PCtηB))+(1-C*C)(γ3(D0PCtηR)-μ(D0PCtηC)) (21)

Now replacing D0PCtηS,0PCDtηE,0PCDtηI,0PCDtηR,0PCDtηD,0PCDtηH,0PCDtηB and D0PCtηC Combining both positive and negative elements with their respective formula results, we have

D˙0PCtηL=θ1-ϕ1. (22)

It can be seen that

Ifθ1>ϕ1thenD˙0PCtηL>0,Ifθ1<ϕ1thenD˙0PCtηL<0,Ifθ1=ϕ1thenD˙0PCtηL=0. (23)

**Remark 1** Incorporating a global stability analysis through Lyapunov functions into our piecewise Caputo Ebola model is a methodological cornerstone. This analysis allows us to explore the system’s behavior across diverse scenarios and parameter ranges. By establishing the global stability of equilibrium points, we attain a profound understanding of how the model responds to various initial conditions and external influences over extended periods. The Lyapunov function serves as a powerful tool in discerning the system’s resilience, providing a comprehensive view of its behavior. This deeper understanding is instrumental in guiding public health strategies, intervention planning, and decision-making processes.

## 5 Analysis of classical piecewise Caputo Ebola virus model

In this section, we explore the existence and uniqueness of the proposed model (7) within a piecewise framework. We aim to establish the existence of a solution and the unique solution property for the considered piecewise differentiable function. To achieve this, we express the system (7) as presented in Lemma 2

D0PCtηℑ(t)=K(t,ℑ(t)),0<α≤1, (24)

and

$$\begin{array}{c} ℑ\left(t\right)=\{\begin{array}{c} {ℑ}_{0}+\int_{0}^{t}K\left(\alpha ,ℑ\left(\alpha \right)\right)d\alpha ,0<t<t_{1},\\ ℑ\left(t_{1}\right)+\frac{1}{\Gamma (\eta )}\int_{0}^{t}{\left(t-\alpha \right)}^{\eta -1}K\left(\alpha ,ℑ\left(\alpha \right)\right)d\alpha ,t_{1}<t<t_{2}. \end{array} \end{array}$$ (25)

where

$$ℑ=\{\begin{array}{c} S(t),\\ E(t),\\ I(t),\\ R(t),\\ D(t),\\ H(t),\\ B(t),\\ C(t). \end{array}{ℑ}_{0}=\{\begin{array}{c} S(0),\\ E(0),\\ I(0),\\ R(0),\\ D(0),\\ H(0),\\ B(0),\\ C(0). \end{array}ℑ(t_{1})=\{\begin{array}{c} S(t_{1}),\\ E(t_{1}),\\ I(t_{1}),\\ R(t_{1}),\\ D(t_{1}),\\ H(t_{1}),\\ B(t_{1}),\\ C(t_{1}). \end{array}$$

K(t,ℑ(t))={K1={ddtK1(S,E,I,R,D,H,B,C),KC1(S,E,I,R,D,H,B,C).K2={ddtK2(S,E,I,R,D,H,B,C),KC2(S,E,I,R,D,H,B,C).K3={ddtK3(S,E,I,R,D,H,B,C),KC3(S,E,I,R,D,H,B,C).K4={ddtK4(S,E,I,R,D,H,B,C),KC4(S,E,I,R,D,H,B,C).K5={ddtK5(S,E,I,R,D,H,B,C),KC5(S,E,I,R,D,H,B,C).K6={ddtK6(S,E,I,R,D,H,B,C),KC6(S,E,I,R,D,H,B,C).K7={ddtK7(S,E,I,R,D,H,B,C),KC7(S,E,I,R,D,H,B,C).K8={ddtK8(S,E,I,R,D,H,B,C),KC8(S,E,I,R,D,H,B,C). (26)

Taking 0 < *t*_1_ ≤ *t*_2_ < ∞ and the norm-containing Banach space *B* = *C*[0, *T*]

$$\begin{array}{c} \|ℑ\|={\text{max}}_{t\in [0,T]}\;|ℑ|. \end{array}$$ (27)

We suppose that *A*_1_ Then there exist *L*_ℑ_ > 0, ∀*P*, ℑ ∈ *B*. We have

$$\begin{array}{c} |K\left(t,ℑ\right)-K\left(t,\tilde{ℑ}\right)|=L_{ℑ}|ℑ-\tilde{ℑ}|. \end{array}$$ (28)

*A*_2_ Then there exist *C*_*K*_ > 0 and *N*_*K*_ > 0, we have

$$\begin{array}{c} |K(t,ℑ(t)|\leq C_{K}|ℑ|+N_{K}. \end{array}$$ (29)

**Theorem 5.1** [45] If function *K* be piecewise continuous on subinterval 0 < *t* ≤ *t*_1_ and *t*_1_ < *t* ≤ *t*_2_ on [0, *T*], also satisfying *A*_2_, then model (7) has at least one solution.

**Proof:** We choose the close subset for both intervals of 0, *T* as *F* of *B* by using Schauder’s theorem

$$\begin{array}{c} F=\{ℑ\in B:\|ℑ\|\leq P_{1,2},P>0\}. \end{array}$$ (30)

Next consider an operator *χ*: *F* → *F* and applying (25) as

$$\begin{array}{c} \chi \left(ℑ\right)=\{\begin{array}{c} {ℑ}_{0}+\int_{0}^{t}K\left(\alpha ,ℑ\left(\alpha \right)\right)d\alpha ,0<t<t_{1},\\ ℑ\left(t_{1}\right)+\frac{1}{\Gamma (\eta )}\int_{0}^{t}{\left(t-\alpha \right)}^{\eta -1}K\left(\alpha ,ℑ\left(\alpha \right)\right)d\alpha ,t_{1}<t<t_{2}. \end{array} \end{array}$$ (31)

On any ℑ ∈ *F*, we have

$$|\chi (ℑ)|\leq \{\begin{array}{c} |{ℑ}_{0}|+\int_{0}^{1}|K(t,ℑ(\alpha ))|d\alpha ,0<t<t_{1},\\ |ℑ(t_{1})|+\frac{1}{\Gamma (\eta )}\int_{0}^{t}{\left(t-\alpha \right)}^{\eta -1}|K(t,ℑ(\alpha ))|d\alpha ,t_{1}<t<t_{2}. \end{array}$$

$$\leq \{\begin{array}{c} |{ℑ}_{0}|+\int_{0}^{t}[C_{K}|ℑ|+N_{K}]d\alpha ,0<t<t_{1},\\ |ℑ(t_{1})|+\frac{1}{\Gamma (\eta )}\int_{0}^{t}{\left(t-\alpha \right)}^{\eta -1}[C_{K}|ℑ|+N_{K}]d\alpha ,t_{1}<t<t_{2}. \end{array}$$

$$\leq \{\begin{array}{c} |{ℑ}_{0}|+t_{1}[C_{K}|ℑ|+N_{K}]=P_{1},0<t<t_{1},\\ |ℑ(t_{1})|+\frac{{\left(t_{2}-t_{1}\right)}^{\eta }}{\Gamma (\eta )}[C_{K}|ℑ|+N_{K}]=P_{2},t_{1}<t<t_{2}. \end{array}$$

$$\leq \{\begin{array}{c} P_{1}\;\;\;\;0<t<t_{1},\\ P_{2}\;\;\;\;t_{1}<t<t_{2}. \end{array}$$

As indicated by the preceding equation given ℑ ∈ *F*, it follows that *χ*(*F*) ⊂ *F*. Consequently, it is demonstrated that the operator *χ* is closed and complete. To further establish the recommended operator’s complete continuity, we consider *t*_*i*_ > *t*_*j*_ ∈ [0, *t*_1_] for the first interval of the integer derivative and assume

$$|\chi \left(ℑ\right)\left(t_{i}\right)-\chi \left(ℑ\right)\left(t_{j}\right)|=|\int_{0}^{t_{i}}K\left(\alpha ,ℑ\left(\alpha \right)\right)d\alpha -\int_{0}^{t_{j}}K(\alpha ,ℑ(\alpha ))d\alpha |,$$

$$\leq \int_{0}^{t_{i}}|K(\alpha ,ℑ(\alpha ))|d\alpha -\int_{0}^{t_{j}}|K(\alpha ,ℑ(\alpha ))|d\alpha ,$$

$$\leq \int_{0}^{t_{i}}[C_{K}|ℑ|+N_{K}]d\alpha -\int_{0}^{t_{j}}[C_{K}|ℑ|+N_{K}]d\alpha ,$$

$$\begin{array}{c} \leq [C_{K}|ℑ|+N_{K}]\left(t_{i}-t_{j}\right). \end{array}$$ (32)

When *t*_*j*_ → *t*_*i*_, then

$$\begin{array}{c} |\chi \left(ℑ\right)\left(t_{i}\right)-\chi \left(ℑ\right)\left(t_{j}\right)|\to 0. \end{array}$$ (33)

Consequently, the equicontinuity of the operator *χ* is demonstrated in [0, *t*_1_]. Consider *t*_*i*_, *t*_*j*_ ∈ [*t*_1_, *t*_2_] in the sense of Caputo as

$$|\chi \left(ℑ\right)\left(t_{i}\right)-\chi \left(ℑ\right)\left(t_{j}\right)|,$$

$$=|\frac{1}{\Gamma (\eta )}\int_{0}^{t_{i}}{\left(t_{i}-\alpha \right)}^{\eta -1}K(\alpha ,ℑ\left(\alpha \right)d\alpha -\int_{0}^{t_{j}}{\left(t_{j}-\alpha \right)}^{\eta -1}K(\alpha ,ℑ\left(\alpha \right)d\alpha |,$$

$$\leq \frac{1}{\Gamma (\eta )}\int_{0}^{t_{j}}[{\left(t_{j}-\alpha \right)}^{\eta -1}-{\left(t_{i}-\alpha \right)}^{\eta -1}]|K(\alpha ,ℑ(\alpha ))|d\alpha +\frac{1}{\Gamma (\eta )}\int_{t_{j}}^{t_{i}}{\left(t_{i}-\alpha \right)}^{\eta -1}|K(\alpha ,ℑ(\alpha ))|d\alpha ,$$

$$\leq \frac{1}{\Gamma (\eta )}[\int_{0}^{t_{j}}\{{\left(t_{j}-\alpha \right)}^{\eta -1}-{\left(t_{i}-\alpha \right)}^{\eta -1}\}d\alpha +\frac{1}{\Gamma (\eta )}\int_{t_{j}}^{t_{i}}{\left(t_{i}-\alpha \right)}^{\eta -1}d\alpha ](C_{K}|ℑ|+N_{K}),$$

$$\leq \frac{\left(C_{K}|ℑ|+N_{K}\right)}{\Gamma (\eta +1)}[t_{i}^{\eta }+t_{j}^{\eta }+2{\left(t_{i}-t_{j}\right)}^{\eta }].$$

If *t*_*j*_ → *t*_*i*_, then

$$|\chi \left(ℑ\right)\left(t_{i}\right)-\chi \left(ℑ\right)\left(t_{j}\right)|\to 0.\;$$

Hence, *χ* exhibits equicontinuity within the interval [*t*_1_, *t*_2_], establishing it as an equicontinuous mapping. By the Arzel-Ascoli theorem, the operator *χ* is completely continuous, uniformly continuous, and bounded. Therefore, in accordance with Schauder’s fixed-point theorem, the piecewise differentiable problem (7) has at least one solution on each sub-interval.

**Theorem 5.2** [45] If *χ* is a contraction operator, the proposed piecewise model has just one root, assuming that *A*_1_ holds.

**Proof** Given that *χ*: *F* → *F* is piecewise continuous, the classical form of ℑ and $\bar{ℑ}\in F$ on [0, *t*_1_]

$$\|\chi \left(ℑ\right)-\chi \bar{(}ℑ)\|={\text{max}}_{t\in [0,t_{1}]}\;|\int_{0}^{t_{i}}K(\alpha ,ℑ(\alpha ))d\alpha -\int_{0}^{t_{i}}K(\alpha ,\bar{ℑ}\left(\alpha \right))d\alpha |,$$

$$\begin{array}{c} \leq t_{1}I_{K}\|ℑ-\bar{ℑ}\|. \end{array}$$ (34)

From Eq (34), we have

$$\begin{array}{c} \|\chi \left(ℑ\right)-\chi \bar{(}ℑ)\|\leq t_{1}I_{K}\|ℑ-\bar{ℑ}\|. \end{array}$$ (35)

*χ* is hence a contraction. According to the Banach fixed-point theorem, the considered model possesses a unique solution within the specified sub-interval. Moreover, for *t* ∈ [*t*_1_, *t*_2_], we have

$$\begin{array}{c} \|\chi \left(ℑ\right)-\chi \bar{(}ℑ)\|={\text{max}}_{t\in [t_{1},t_{2}]}\;|\frac{1}{\Gamma (\eta )}\int_{t_{1}}^{t_{2}}{\left(t-\alpha \right)}^{\eta -1}K(\alpha ,ℑ(\alpha ))d\alpha -\frac{1}{\Gamma (\eta )}\int_{t_{1}}^{t_{2}}{\left(t-\alpha \right)}^{\eta -1}K(\alpha ,\bar{ℑ}\left(\alpha \right))d\alpha |, \end{array}$$

$$\begin{array}{c} \leq \frac{{\left(t_{2}-t_{1}\right)}^{\eta }}{\Gamma (\eta -1)}I_{K}\|ℑ-\bar{ℑ}\|. \end{array}$$ (36)

From Eq (36), we have

$$\begin{array}{c} \|\chi \left(ℑ\right)-\chi \bar{(}ℑ)\|\leq \frac{{\left(t_{2}-t_{1}\right)}^{\eta }}{\Gamma (\eta -1)}I_{K}\|ℑ-\bar{ℑ}\|. \end{array}$$ (37)

Consequently, *χ* is a contraction implying that the proposed piecewise model has at most one solution.

## 6 Numerical scheme

This section derives the numerical strategy for the model that we propose (7). The piecewise integration for classical and Caputo format is applied to Eq (7) in the following way:

$$\begin{array}{c} \begin{array}{c} S\left(t\right)=\{\begin{array}{c} S_{0}+\int_{0}^{t_{1}}K_{1}\left(\alpha ,S\right)d\alpha ,0<t\leq t_{1}\\ S\left(t_{1}\right)+\frac{1}{\Gamma (\eta )}\int_{t_{1}}^{t_{2}}{\left(t-\alpha \right)}^{\eta -1}K_{1}\left(\alpha ,S\right)d\alpha ,t_{1}<t\leq t_{2} \end{array}\\ E\left(t\right)=\{\begin{array}{c} E_{0}+\int_{0}^{t_{1}}K_{2}\left(\alpha ,E\right)d\alpha ,0<t\leq t_{1}\\ E\left(t_{1}\right)+\frac{1}{\Gamma (\eta )}\int_{t_{1}}^{t_{2}}{\left(t-\alpha \right)}^{\eta -1}K_{2}\left(\alpha ,E\right)d\alpha ,t_{1}<t\leq t_{2} \end{array}\\ I\left(t\right)=\{\begin{array}{c} I_{0}+\int_{0}^{t_{1}}K_{3}\left(\alpha ,I\right)d\alpha ,0<t\leq t_{1}\\ I\left(t_{1}\right)+\frac{1}{\Gamma (\eta )}\int_{t_{1}}^{t_{2}}{\left(t-\alpha \right)}^{\eta -1}K_{3}\left(\alpha ,I\right)d\alpha ,t_{1}<t\leq t_{2} \end{array}\\ R\left(t\right)=\{\begin{array}{c} R_{0}+\int_{0}^{t_{1}}K_{4}\left(\alpha ,R\right)d\alpha ,0<t\leq t_{1}\\ R\left(t_{1}\right)+\frac{1}{\Gamma (\eta )}\int_{t_{1}}^{t_{2}}{\left(t-\alpha \right)}^{\eta -1}K_{4}\left(\alpha ,R\right)d\alpha ,t_{1}<t\leq t_{2} \end{array}\\ D\left(t\right)=\{\begin{array}{c} D_{0}+\int_{0}^{t_{1}}K_{5}\left(\alpha ,D\right)d\alpha ,0<t\leq t_{1}\\ D\left(t_{1}\right)+\frac{1}{\Gamma (\eta )}\int_{t_{1}}^{t_{2}}{\left(t-\alpha \right)}^{\eta -1}K_{5}\left(\alpha ,D\right)d\alpha ,t_{1}<t\leq t_{2} \end{array}\\ H\left(t\right)=\{\begin{array}{c} H_{0}+\int_{0}^{t_{1}}K_{6}\left(\alpha ,H\right)d\alpha ,0<t\leq t_{1}\\ H\left(t_{1}\right)+\frac{1}{\Gamma (\eta )}\int_{t_{1}}^{t_{2}}{\left(t-\alpha \right)}^{\eta -1}K_{6}\left(\alpha ,H\right)d\alpha ,t_{1}<t\leq t_{2} \end{array}\\ B\left(t\right)=\{\begin{array}{c} B_{0}+\int_{0}^{t_{1}}K_{7}\left(\alpha ,B\right)d\alpha ,0<t\leq t_{1}\\ B\left(t_{1}\right)+\frac{1}{\Gamma (\eta )}\int_{t_{1}}^{t_{2}}{\left(t-\alpha \right)}^{\eta -1}K_{7}\left(\alpha ,B\right)d\alpha ,t_{1}<t\leq t_{2} \end{array}\\ C\left(t\right)=\{\begin{array}{c} C_{0}+\int_{0}^{t_{1}}K_{8}\left(\alpha ,C\right)d\alpha ,0<t\leq t_{1}\\ C\left(t_{1}\right)+\frac{1}{\Gamma (\eta )}\int_{t_{1}}^{t_{2}}{\left(t-\alpha \right)}^{\eta -1}K_{8}\left(\alpha ,C\right)d\alpha ,t_{1}<t\leq t_{2} \end{array} \end{array} \end{array}$$ (38)

Now, we formulate the numerical technique for one of the equations in problem (38) and the remaining equations can be derived in a similar manner. At *t* = *t*_*n*+1_

$$\begin{array}{c} S\left(t\right)=\{\begin{array}{c} S_{0}+\int_{0}^{t_{1}}K_{1}\left(S,E,I,R,D,H,B,C,\alpha \right)d\alpha ,0<t\leq t_{1},\\ S\left(t_{1}\right)+\frac{1}{\Gamma (\eta )}\int_{t_{1}}^{t_{n+1}}{\left(t-\alpha \right)}^{\eta -1}K_{1}\left(S,E,I,R,D,H,B,C,\alpha \right)d\alpha ,t_{1}<t\leq t_{2}, \end{array} \end{array}$$ (39)

and

$$S(t_{n+1})=\{\begin{array}{c} S_{0}+\sum_{m=0}^{j}\int_{t_{m}}^{t_{m+1}}K_{1}(\left(S,E,I,R,D,H,B,C,\alpha \right))d\alpha \\ S(t_{1})+\frac{1}{\Gamma (\eta )}\sum_{m=j+1}^{n}\int_{t_{m}}^{t_{m+1}}{\left(t-\alpha \right)}^{\eta -1}K_{1}(\left(S,E,I,R,D,H,B,C,\alpha \right))d\alpha \end{array}$$

Utilising the Newton approximation, we approximate *K*_1_(*t*, *S*, *E*, *I*, *R*, *D*, *H*, *B*, *C*,) within [*t*_*m*_, *t*_*m*_ + 1]

$$\begin{array}{cc} K_{1}\left(t,S,E,I,R,D,H,B,C\right)=P_{m}\left(t\right) & ≃\;K_{1}\left(t_{m-2},S_{m-2},E_{m-2},I_{m-2},R_{m-2},D_{m-2},H_{m-2},B_{m-2},C_{m-2}\right)\\ & +\frac{1}{\Delta t}[K_{1}\left(t_{m-1},S_{m-1},E_{m-1},I_{m-1},R_{m-1},D_{m-1},H_{m-1},B_{m-1},C_{m-1}\right)\\ & -K_{1}\left(t_{m-2},S_{m-2},E_{m-2},I_{m-2},R_{m-2},D_{m-2},H_{m-2},B_{m-2},C_{m-2}\right)]\\ & \times \left(\alpha -t_{m-2}\right)+\frac{1}{2{\left(\Delta t\right)}^{2}}[K_{1}\left(t_{m},S_{m},E_{m},I_{m},R_{m},D_{m},H_{m},B_{m},C_{m}\right)\\ & -2K_{1}\left(t_{m-1},S_{m-1},E_{m-1},I_{m-1},R_{m-1},D_{m-1},H_{m-1},B_{m-1},C_{m-1}\right)\\ & -K_{1}\left(t_{m-2},S_{m-2},E_{m-2},I_{m-2},R_{m-2},D_{m-2},H_{m-2},B_{m-2},C_{k-2}\right)]\\ & \times \left(\alpha -t_{m-2}\right)\left(\alpha -t_{m-1}\right) \end{array}$$ (40)

Replacing *K*_1_(*α*, *S*, *E*, *I*, *R*, *D*, *H*, *B*, *C*) by *P*_*m*_(*t*) within [*t*_*m*_, *t*_*m*_ + 1] integrating, we have following scheme

$$\begin{array}{c} S\left(t_{n+1}\right)=\{\begin{array}{c} S_{0}+\sum_{m=2}^{j}[\begin{array}{c} \frac{5}{12}K_{1}\left(P_{2},t_{m-2}\right)\Delta t\\ -\frac{4}{3}K_{1}\left(P_{1},t_{m-1}\right)\Delta t+K_{1}\left(P,t_{k}\right) \end{array}],\\ S\left(t_{1}\right)+\{\begin{array}{c} \frac{{\left(\Delta t\right)}^{\eta -1}}{\Gamma (\eta +1)}\sum_{m=j+3}^{n}[K_{1}\left(P_{2},t_{m-2}\right)]\hbar \\ +\frac{{\left(\Delta t\right)}^{\eta -1}}{\Gamma (\eta +2)}\sum_{m=j+3}^{n}[\begin{array}{c} K_{1}\left(P_{1},t_{m-1}\right)\\ -K_{1}\left(P_{2},t_{m-2}\right) \end{array}]\psi \\ +\frac{{\left(\Delta t\right)}^{\eta -1}}{2\Gamma (\eta +3)}\sum_{m=j+3}^{n}[\begin{array}{c} K_{1}\left(P,t_{m}\right)-2K_{1}\left(P_{1},t_{m-1}\right)\\ +K_{1}\left(P_{2},t_{m-2}\right) \end{array}]\delta \end{array}\}, \end{array} \end{array}$$ (41)

For the remaining seven compartments, we can write the Newton interpolation approximation as follows:

$$\begin{array}{c} E\left(t_{n+1}\right)=\{\begin{array}{c} E_{0}+\sum_{m=2}^{j}[\begin{array}{c} \frac{5}{12}K_{2}\left(P_{2},t_{m-2}\right)\Delta t\\ -\frac{4}{3}K_{2}\left(P_{1},t_{m-1}\right)\Delta t+K_{2}\left(P,t_{k}\right) \end{array}],\\ E\left(t_{1}\right)+\{\begin{array}{c} \frac{{\left(\Delta t\right)}^{\eta -1}}{\Gamma (\eta +1)}\sum_{m=j+3}^{n}[K_{2}\left(P_{2},t_{m-2}\right)]\hbar \\ +\frac{{\left(\Delta t\right)}^{\eta -1}}{\Gamma (\eta +2)}\sum_{m=j+3}^{n}[\begin{array}{c} K_{2}\left(P_{1},t_{m-1}\right)\\ -K_{2}\left(P_{2},t_{m-2}\right) \end{array}]\psi \\ +\frac{{\left(\Delta t\right)}^{\eta -1}}{2\Gamma (\eta +3)}\sum_{m=j+3}^{n}[\begin{array}{c} K_{2}\left(P,t_{m}\right)-2K_{2}\left(P_{1},t_{m-1}\right)\\ +K_{2}\left(P_{2},t_{m-2}\right) \end{array}]\delta \end{array}\}, \end{array} \end{array}$$ (42)

$$\begin{array}{c} I\left(t_{n+1}\right)=\{\begin{array}{c} I_{0}+\sum_{m=2}^{j}[\begin{array}{c} \frac{5}{12}K_{3}\left(P_{2},t_{m-2}\right)\Delta t\\ -\frac{4}{3}K_{3}\left(P_{1},t_{m-1}\right)\Delta t+K_{3}\left(P,t_{k}\right) \end{array}],\\ I\left(t_{1}\right)+\{\begin{array}{c} \frac{{\left(\Delta t\right)}^{\eta -1}}{\Gamma (\eta +1)}\sum_{m=j+3}^{n}[K_{3}\left(P_{2},t_{m-2}\right)]\hbar \\ +\frac{{\left(\Delta t\right)}^{\eta -1}}{\Gamma (\eta +2)}\sum_{m=j+3}^{n}[\begin{array}{c} K_{3}\left(P_{1},t_{m-1}\right)\\ -K_{3}\left(P_{2},t_{m-2}\right) \end{array}]\psi \\ +\frac{{\left(\upsilon t\right)}^{\eta -1}}{2\Gamma (\eta +3)}\sum_{m=j+3}^{n}[\begin{array}{c} K_{3}\left(P,t_{m}\right)-2K_{3}\left(P_{1},t_{m-1}\right)\\ +K_{3}\left(P_{2},t_{m-2}\right) \end{array}]\delta \end{array}\}, \end{array} \end{array}$$ (43)

$$\begin{array}{c} R\left(t_{n+1}\right)=\{\begin{array}{c} R_{0}+\sum_{m=2}^{j}[\begin{array}{c} \frac{5}{12}K_{4}\left(P_{2},t_{m-2}\right)\Delta t\\ -\frac{4}{3}K_{4}\left(P_{1},t_{m-1}\right)\Delta t+K_{4}\left(P,t_{k}\right) \end{array}],\\ R\left(t_{1}\right)+\{\begin{array}{c} \frac{{\left(\Delta t\right)}^{\eta -1}}{\Gamma (\eta +1)}\sum_{m=j+3}^{n}[K_{4}\left(P_{2},t_{m-2}\right)]\hbar \\ +\frac{{\left(\Delta t\right)}^{\eta -1}}{\Gamma (\eta +2)}\sum_{m=j+3}^{n}[\begin{array}{c} K_{4}\left(P_{1},t_{m-1}\right)\\ -K_{4}\left(P_{2},t_{m-2}\right) \end{array}]\psi \\ +\frac{{\left(\Delta t\right)}^{\eta -1}}{2\Gamma (\eta +3)}\sum_{m=j+3}^{n}[\begin{array}{c} K_{4}\left(P,t_{m}\right)-2K_{4}\left(P_{1},t_{m-1}\right)\\ +K_{4}\left(P_{2},t_{m-2}\right) \end{array}]\delta \end{array}\}, \end{array} \end{array}$$ (44)

$$\begin{array}{c} D\left(t_{n+1}\right)=\{\begin{array}{c} D_{0}+\sum_{m=2}^{j}[\begin{array}{c} \frac{5}{12}K_{5}\left(P_{2},t_{m-2}\right)\Delta t\\ -\frac{4}{3}K_{5}\left(P_{1},t_{m-1}\right)\Delta t+K_{5}\left(P,t_{k}\right) \end{array}],\\ D\left(t_{1}\right)+\{\begin{array}{c} \frac{{\left(\Delta t\right)}^{\eta -1}}{\Gamma (\eta +1)}\sum_{m=j+3}^{n}[K_{5}\left(P_{2},t_{m-2}\right)]\hbar \\ +\frac{{\left(\Delta t\right)}^{\eta -1}}{\Gamma (\eta +2)}\sum_{m=j+3}^{n}[\begin{array}{c} K_{5}\left(P_{1},t_{m-1}\right)\\ -K_{5}\left(P_{2},t_{m-2}\right) \end{array}]\psi \\ +\frac{{\left(\upsilon t\right)}^{\eta -1}}{2\Gamma (\eta +3)}\sum_{m=j+3}^{n}[\begin{array}{c} K_{5}\left(P,t_{m}\right)-2K_{5}\left(P_{1},t_{m-1}\right)\\ +K_{5}\left(P_{2},t_{m-2}\right) \end{array}]\delta \end{array}\}, \end{array} \end{array}$$ (45)

$$\begin{array}{c} H\left(t_{n+1}\right)=\{\begin{array}{c} H_{0}+\sum_{m=2}^{j}[\begin{array}{c} \frac{5}{12}K_{6}\left(P_{2},t_{m-2}\right)\Delta t\\ -\frac{4}{3}K_{6}\left(P_{1},t_{m-1}\right)\Delta t+K_{6}\left(P,t_{k}\right) \end{array}],\\ H\left(t_{1}\right)+\{\begin{array}{c} \frac{{\left(\upsilon t\right)}^{\eta -1}}{\Gamma (\eta +1)}\sum_{m=j+3}^{n}[K_{6}\left(P_{2},t_{m-2}\right)]\hbar \\ +\frac{{\left(\Delta t\right)}^{\eta -1}}{\Gamma (\eta +2)}\sum_{m=j+3}^{n}[\begin{array}{c} K_{6}\left(P_{1},t_{m-1}\right)\\ -K_{6}\left(P_{2},t_{m-2}\right) \end{array}]\psi \\ +\frac{{\left(\Delta t\right)}^{\eta -1}}{2\Gamma (\eta +3)}\sum_{m=j+3}^{n}[\begin{array}{c} K_{6}\left(P,t_{m}\right)-2K_{6}\left(P_{1},t_{m-1}\right)\\ +K_{6}\left(P_{2},t_{m-2}\right) \end{array}]\delta \end{array}\}, \end{array} \end{array}$$ (46)

$$\begin{array}{c} B\left(t_{n+1}\right)=\{\begin{array}{c} B_{0}+\sum_{m=2}^{j}[\begin{array}{c} \frac{5}{12}K_{7}\left(P_{2},t_{m-2}\right)\Delta t\\ -\frac{4}{3}K_{7}\left(P_{1},t_{m-1}\right)\Delta t+K_{7}\left(P,t_{k}\right) \end{array}],\\ B\left(t_{1}\right)+\{\begin{array}{c} \frac{{\left(\Delta t\right)}^{\eta -1}}{\Gamma (\eta +1)}\sum_{m=j+3}^{n}[K_{7}\left(P_{2},t_{m-2}\right)]\hbar \\ +\frac{{\left(\Delta t\right)}^{\eta -1}}{\Gamma (\eta +2)}\sum_{m=j+3}^{n}[\begin{array}{c} K_{7}\left(P_{1},t_{m-1}\right)\\ -K_{7}\left(P_{2},t_{m-2}\right) \end{array}]\psi \\ +\frac{{\left(\Delta t\right)}^{\eta -1}}{2\Gamma (\eta +3)}\sum_{m=j+3}^{n}[\begin{array}{c} K_{7}\left(P,t_{m}\right)-2K_{7}\left(P_{1},t_{m-1}\right)\\ +K_{7}\left(P_{2},t_{m-2}\right) \end{array}]\delta \end{array}\}, \end{array} \end{array}$$ (47)

$$\begin{array}{c} C\left(t_{n+1}\right)=\{\begin{array}{c} C_{0}+\sum_{m=2}^{j}[\begin{array}{c} \frac{5}{12}K_{8}\left(P_{2},t_{m-2}\right)\Delta t\\ -\frac{4}{3}K_{8}\left(P_{1},t_{m-1}\right)\Delta t+K_{8}\left(P,t_{k}\right) \end{array}],\\ C\left(t_{1}\right)+\{\begin{array}{c} \frac{{\left(\Delta t\right)}^{\eta -1}}{\Gamma (\eta +1)}\sum_{m=j+3}^{n}[K_{8}\left(P_{2},t_{m-2}\right)]\hbar \\ +\frac{{\left(\Delta t\right)}^{\eta -1}}{\Gamma (\eta +2)}\sum_{m=j+3}^{n}[\begin{array}{c} K_{8}\left(P_{1},t_{m-1}\right)\\ -K_{8}\left(P_{2},t_{m-2}\right) \end{array}]\psi \\ +\frac{{\left(\Delta t\right)}^{\eta -1}}{2\Gamma (\eta +3)}\sum_{m=j+3}^{n}[\begin{array}{c} K_{8}\left(P,t_{m}\right)-2K_{8}\left(P_{1},t_{m-1}\right)\\ +K_{8}\left(P_{2},t_{m-2}\right) \end{array}]\delta \end{array}\}, \end{array} \end{array}$$ (48)

where

$$P=S^{m},E^{m},I^{m},R^{m},D^{m},H^{m},B^{m},C^{m}$$

$$P_{1}=S^{m-1},E^{m-1},I^{m-1},R^{m-1},D^{m-1},H^{m-1},B^{m-1},C^{m-1}$$

$$P_{2}=S^{m-2},E^{m-2},I^{m-2},R^{m-2},D^{m-2},H^{m-2},B^{m-2},C^{m-2}$$

and

$$\delta =[\begin{array}{c} {\left(1+n-m\right)}^{\eta }(2{\left(n-m\right)}^{2}+(3\eta +10)(n-m)+2\eta^{2}+9\eta +12)\\ -(n-m)(2{\left(n-m\right)}^{2}+(5\eta +10)(n-m)+6\eta^{2}+18\eta +12) \end{array}],$$

$$\hbar =[{\left(1+n-m\right)}^{\eta }(3+2\eta -m+n)-(n-m)(3+3\eta -m+n)],$$

$$\psi =[{\left(1+n-m\right)}^{\eta }-(n-m)].$$

**Fig 18:**
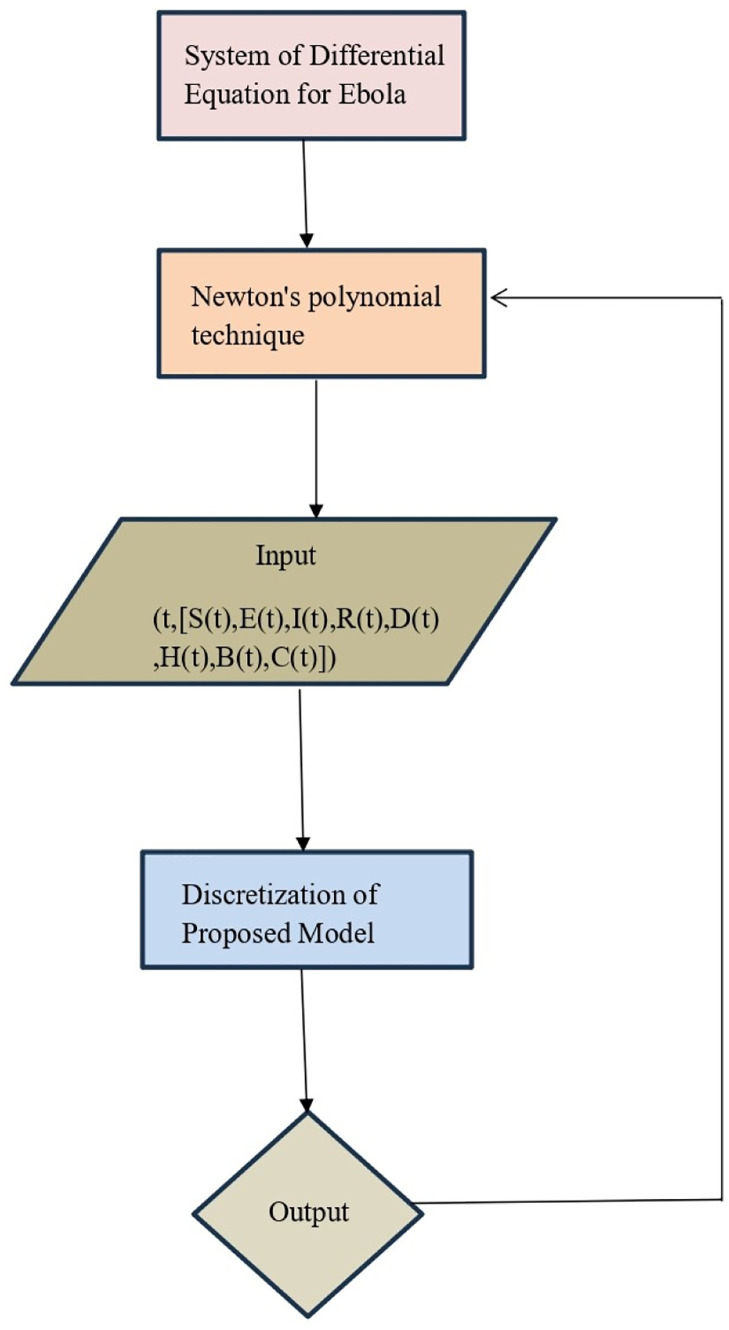
Bayesian algorithm’s network type flow chart.

Fig 18 dipicted the Bayesian algorithm’s network type flow chart of above methdology.

## 7 Results of proposed scheme

**Fig 19:**
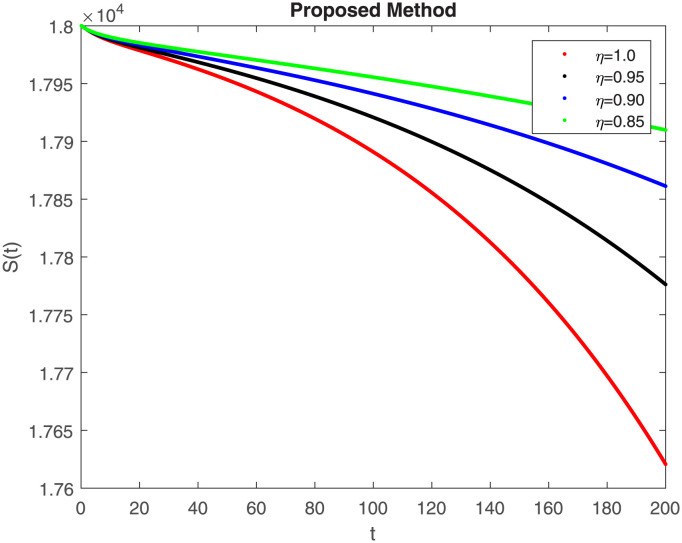
Dynamics of S(t) at different fractional order with proposed piecewise caputo operator.

**Fig 20:**
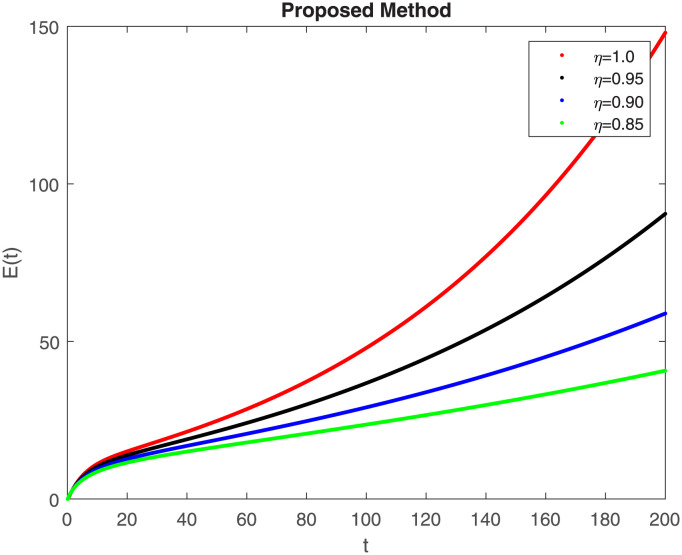
Dynamics of E(t) at different fractional order with proposed piecewise caputo operator.

**Fig 21:**
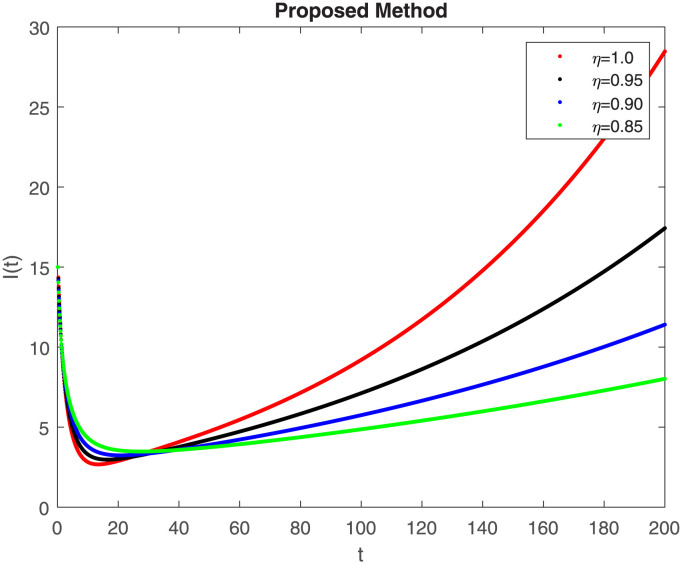
Dynamics of I(t) at different fractional order with proposed piecewise caputo operator.

**Fig 22:**
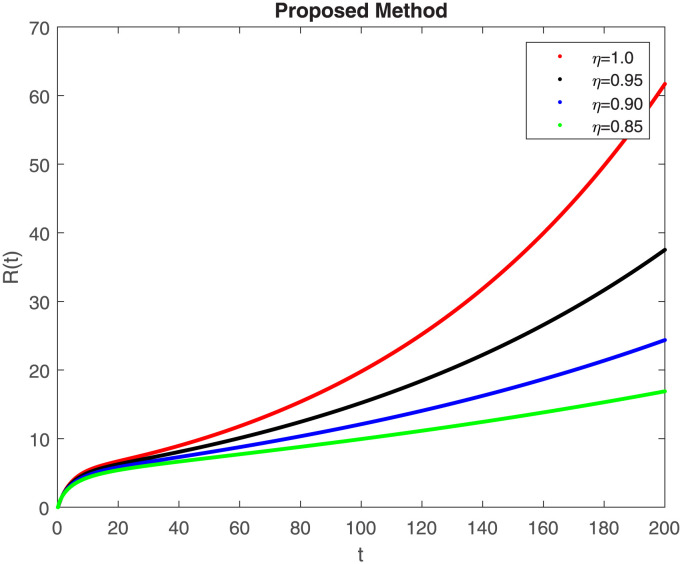
Dynamics of R(t) at different fractional order with proposed piecewise caputo operator.

**Fig 23:**
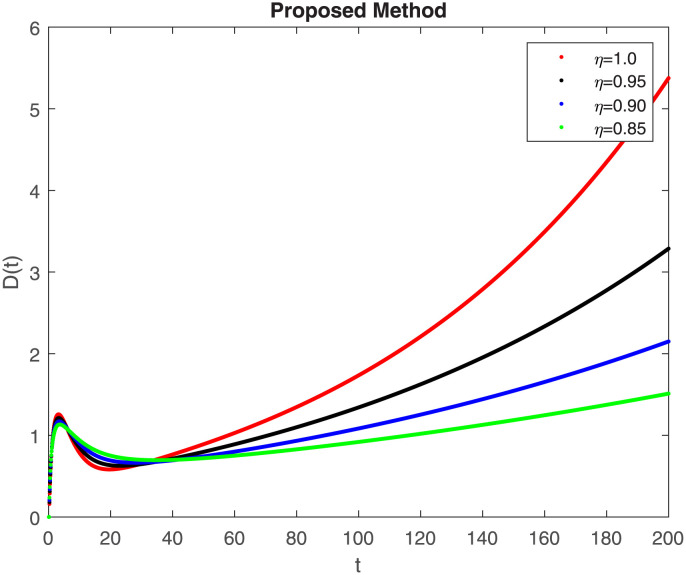
Dynamics of D(t) at different fractional order with proposed piecewise caputo operator.

**Fig 24:**
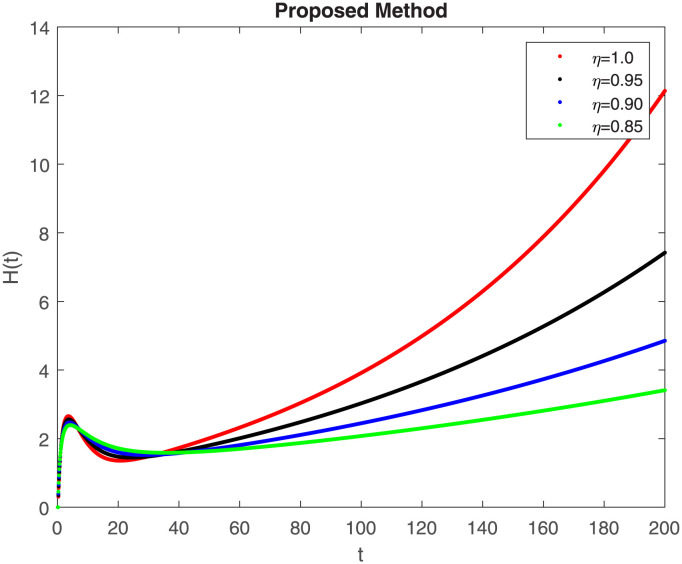
Dynamics of H(t) at different fractional order with proposed piecewise caputo operator.

**Fig 25:**
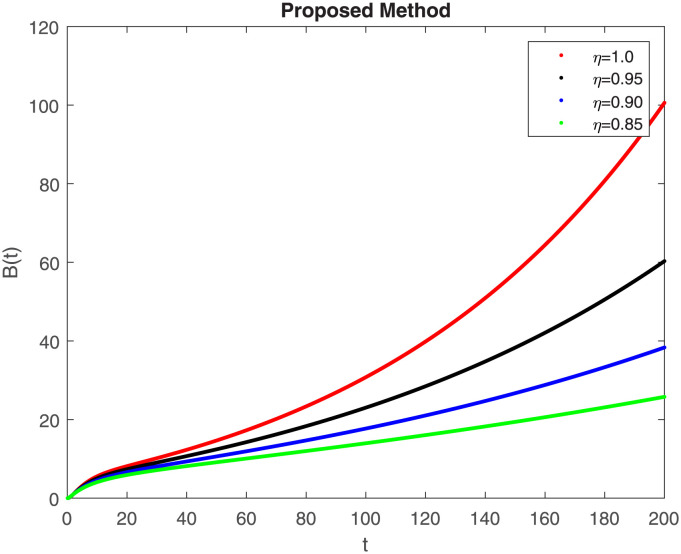
Dynamics of B(t) at different fractional order with proposed piecewise caputo operator.

**Fig 26:**
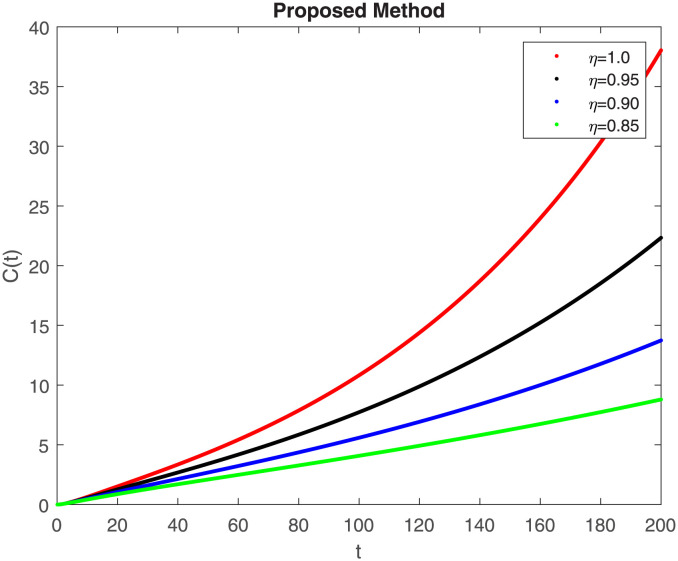
Dynamics of C(t) at different fractional order with proposed piecewise caputo operator.

The numerical simulation utilizing the derived method is presented in this section, incorporating the concepts of both classical and piecewise derivatives. The results are illustrated in Figs 19 to 26. The initial values and parameters for systems (7) are listed in Table 1 and were taken from [43]. Using data from Table 1 that correspond to various fractional orders, for S, E, I, R, D, H, B, and C, we simulate how the solution to model (7) behaves. We have considered the two subintervals †_1_ = [0, *t*_1_] = [0, 40] and †_2_ = [*t*_1_, *T*] = [40, 200] for the simulation purpose of Figs 19 to 26. With the higher susceptibility predicted by the Caputo operator, Fig 19 illustrates the increasing trend of the susceptible population for decreasing values of *η*. A distinct crossover behavior of the susceptible population is observed at time 40. Fig 20 depicts the decreasing behavior of the exposed population for decreasing values of *η*. Similar behavior can be observed in the populations that are infected, asymptomatic but still infectious, dead but not buried, hospitalized, and buried, as shown in Figs 21–25 correspondingly. Fig 21 depicts, initially the infected individual decreases after t = 10 the infected individual increases a clear cross behavior observed at the time 40. In Fig 26 we can observed, fully recovered populations begin to rise as fractional values rise. A distinct crossover behavior of the fully recovered individuals is noticeable at the time 40.

These findings underscore the substantial impact of varying *η* values on different compartments within the epidemic model. A smaller fractional order usually leads to a quicker decay process and a slower growth process. Conversely, a larger fractional order results in a faster growth process and a slower decay process. The clear crossovers observed at time 40 across various population states signify critical junctures in the dynamics, potentially indicating shifts in disease spread, recovery rates, or other key epidemiological parameters. Understanding these dynamics provides valuable insights into the complex behavior of the epidemic under the influence of fractional orders, offering potential clues for intervention strategies and disease control measures. The persistence of the disease in the population significantly influences the analysis of mathematical models. This model assumes that the population volume is stable throughout time and offers a reliable estimate for brief periods with similar populations. Here we can also observe the memory effect of fractional order derivatives in simulations as compared to ordinary derivatives in this model in different time intervals.

Some authors have previously examined the issue of fractional order problem in relation to the administration of vaccines for the Ebola disease, but always under the assumption of an unlimited supply of vaccines. It transpires that the resolution to this mathematical quandary is evident: the solution entails vaccinating all susceptible individuals at the onset of the outbreak. In the event that vaccines are accessible without any limitations, it would be feasible to completely eradicate Ebola within a brief time frame. These findings underscore the significance of an effective Ebola vaccine and the highly favorable outcomes that can be attained if the quantity of available vaccines meets the population’s requirements. Regrettably, such a scenario is not plausible: in the event that an effective Ebola vaccine materializes, there will invariably be restrictions on the quantity of available vaccines, as well as constraints governing their proper administration within a short timeframe; economic considerations may also arise.

## 8 Conclusion

In this study, we used the piecewise operator in the form of classical and Caputo operators to analyze the behavior of the Ebola virus model. The existence and uniqueness of a solution bearing a piecewise derivative are investigated for the disease model given earlier. The sensitivity analysis indicates that the value *R*_0_ directly proportional to the per capita rate at which exposed individuals become infectious (*σ*), contact rate of infective and susceptible individuals (*β*_*i*_), contact rate of infective and dead individuals (*β*_*d*_), contact rate of infective and hospitalized individuals (*β*_*h*_), contact rate of infective and asymptomatic individuals (*β*_*r*_) and per capita rate of progression of individuals from the dead class to the buried class (*δ*_1_). These factors are adjustable through the efficient implementation of vaccination campaigns. The system is locally and globally stable and also to identify waves, the second derivative of the Lyapunov function was used to determine the sign of the second derivative of each class. To approximate the solution to the stated issue, the piecewise Newton polynomial method is utilized. Moreover, the study has presented outcomes that were compared with real-world data about individuals reported as infected. Instances of abrupt shifts in their state of rest or uniform motion, commonly referred to as crossover behavior, are prevalent in various real-world scenarios. Traditional derivatives, whether fractional or classical, often fall short of adequately capturing this phenomenon. The piecewise derivatives of fractional order, as demonstrated in the numerical findings, offer a more comprehensive depiction of the proposed model compared to traditional integer-order epidemic models. The graphical confirmation of the equilibrium point’s continuity and boundedness is established by employing specific initial conditions and parameters. Thus, the suggested approach is crucial for studying epidemiological models. Figures also illustrate the significance of the fractional piecewise operator with different time intervals as classical as well as fractional values which are helpful for planning, and decision-making to control the disease in society. We have studied a fractional order problem with state and control restrictions for the first time in the Ebola literature. It is a mathematical representation of a public health problem with a limited supply of vaccines. The findings provide important information on how many vaccines should be purchased to reduce the number of new illnesses at the lowest possible cost.

In conclusion, while the piecewise Caputo derivative is a valuable tool for modeling epidemic dynamics, it comes with limitations. The challenge of accurately identifying transition points or intervals where the model behavior shifts can impact its accuracy. These difficulties may hinder the precise modeling of abrupt changes in epidemic scenarios. Therefore, despite its advantages, careful consideration of these limitations is essential when applying the piecewise Caputo derivative in epidemic modeling. This research lays the groundwork for future applications in more intricate dynamical scenarios, including derivatives of the fractal-fractional and Mittag-Leffler types. Additionally, the model will be subject to examination under stochastic fractional-order differential equations in the future, incorporating optimal control procedures and non-singular differential operators.
